# Human circulating small non-coding RNA signature as a non-invasive biomarker in clinical diagnosis of acute myeloid leukaemia

**DOI:** 10.7150/thno.80054

**Published:** 2023-02-13

**Authors:** Lin Xia, Huanping Guo, Xiao Wu, Yinying Xu, Pan Zhao, Bingbing Yan, Yunjing Zeng, Yundi He, Dan Chen, Robert Peter Gale, Yunfang Zhang, Xi Zhang

**Affiliations:** 1 Medical Center of Hematology, Xinqiao Hospital, State Key Laboratory of Trauma, Burns and Combined Injury, Army Medical University, Chongqing, China.; 2 Haematology Centre, Department of Immunology and Inflammation, Imperial College London, London, UK.; 3 Clinical and Translational Research Center of Shanghai First Maternity and Infant Hospital, Shanghai Key Laboratory of Signaling and Disease Research, Frontier Science Center for Stem Cell Research, School of Life Sciences and Technology, Tongji University, Shanghai, China.; 4 Jinfeng Laboratory, Chongqing, China

**Keywords:** human circulating sncRNA, tsRNA, ysRNA, rsRNA, alternative noninvasive biomarker

## Abstract

**Background:** Acute myeloid leukaemia (AML) is the most common acute leukaemia in adults; AML is highly heterogeneous and involves abnormalities at multiple omics levels. Small non-coding RNAs (sncRNAs) present in body fluids are important regulatory molecules and considered promising non-invasive clinical diagnostic biomarkers for disease. However, the signature of sncRNA profile alteration in AML patient serum and bone marrow supernatant is still under exploration.

**Methods:** We examined data for blood and bone marrow samples from 80 consecutive, newly-diagnosed patients with AML and 12 healthy controls for high throughput small RNA-sequencing. Differentially expressed sncRNAs were analysed to reveal distinct patterns between AML patients and controls. Machine learning methods were used to evaluate the efficiency of specific sncRNAs in discriminating individuals with AML from controls. The altered expression level of individual sncRNAs was evaluated by RT-PCR, Q-PCR, and northern blot. Correlation analysis was employed to assess sncRNA patterns between serum and bone marrow supernatant.

**Results:** We identified over 20 types of sncRNA categories beyond miRNAs in both serum and bone marrow supernatant, with highly coordinated expression patterns between them. Non-classical sncRNAs, including rsRNA (62.86%), ysRNA (14.97%), and tsRNA (4.22%), dominated among serum sncRNAs and showed sensitive alteration patterns in AML patients. According to machine learning-based algorithms, the tsRNA-based signature robustly discriminated subjects with AML from controls and was more reliable than that comprising miRNAs. Our data also showed that serum tsRNAs to be closely associated with AML prognosis, suggesting the potential application of serum tsRNAs as biomarkers to assist in AML diagnosis.

**Conclusions:** We comprehensively characterized the expression pattern of circulating sncRNAs in blood and bone marrow and their alteration signature between healthy controls and AML patients. This study enriches research of sncRNAs in the regulation of AML, and provides insights into the role of sncRNAs in AML.

## Introduction

Small non-coding RNAs (sncRNAs), including microRNAs (miRNAs), tRNA-derived small RNAs (tsRNAs), rRNA-derived small RNAs (rsRNAs), and YRNA-derived small RNAs (ysRNAs), are important regulatory molecules 1-3. sncRNAs regulate multiple aspects of mRNA stability, translation efficiency, and ribosome biogenesis 4-9. Dysregulation of blood miRNAs has been reported in breast and liver cancers, among others 10, 11. Common methods to quantify blood sncRNAs use miRNA arrays or small RNA-seq with nucleotides in the range of 18-30 nt during sequencing, resulting in the loss of data for small RNAs of greater nucleotide lengths, such as piRNAs (~30 nt), tsRNAs (29-34 nt), and rsRNAs (> 30 nt) 12. By expanding the nucleotide length to 45 nt, we report that tsRNAs are highly enriched in vertebrate blood in stable forms and conserved in diverse species, from fish to humans. Blood concentrations of tsRNAs change after acute inflammation induced by lipopolysaccharide (LPS) injection in mice and monkeys and after hepatitis-B-virus (HBV) infection in humans, suggesting roles in the inflammation response 13.

Acute myeloid leukaemia (AML) has a heterogeneous mutation topography and involves diverse non-coding RNA alterations 14. The roles of miRNAs in AML have been broadly explored in recent years, and unmistakable expression profiles of miRNA have been distinguished in leukaemia 15-18. Due to the attractive advantages of circulating miRNAs, such as sensitivity, stability, and non-invasiveness, numerous studies have reported that circulating miRNAs can serve as a novel class of non-invasive diagnostic and prognostic biomarkers in multiple cancers, with promising clinical application prospects 19-23. However, most of those studies focused on miRNAs, with scant research into other types of circulating sncRNA in serum. Therefore, in this study, we examined sncRNAs in 122 blood and bone marrow samples from 80 consecutive subjects with newly-diagnosed, untreated AML to quantify sncRNAs, consider their potential role in AML biology, and identify the AML-specific alteration pattern of circulating sncRNAs as non-invasive biomarkers to assist in diagnosis.

## Materials and Methods

### Ethics committee approval and patient consent

All experiments were performed in accordance with the principles set out in the World Medical Association Declaration of Helsinki. This study was approved by the Institutional Ethics Committees of Xinqiao Hospital, and informed consent was signed by each participant.

### Clinical data and sample collection

We studied blood and bone marrow samples from 80 consecutive subjects with acute myeloid leukaemia (AML) at the Medical Center of Hematology, Xinqiao Hospital from 2018 to 2021 (**Table S1**). Blood samples (5 ml) were collected from 80 AML patients and 12 healthy controls; bone marrow samples (5 ml) were collected from 30 AML patients and prepared for RNA extraction and small RNA high-throughput sequencing. The subjects gave written informed consent consistent with the precepts of the Declaration of Helsinki. The study was approved by the Institutional Ethics Committees of Xinqiao Hospital.

### Sample isolation and RNA extraction

Samples were incubated at 24 °C for 30 min and then centrifuged twice (2,000/8,500 × g) at 4 °C for 10 min. RNA was extracted by using TRIzol LS reagent (Invitrogen, Carlsbad, CA, USA) according to the manufacturer's protocol. One microlitre of cel-miR-39 was added to the TRIzol LS mixtures as a spike-in control before serum RNA extraction.

### Small RNA high-throughput sequencing and data processing

Small RNA library construction and sequencing were performed by BGI (Shenzhen, Guangdong, China) using NEB Small RNA Sample Pre Kit (NEB) and UMI Small RNA library construction methods. Raw sequencing reads were processed using SPORTS software 24. Reads were aligned to the human reference genome, miRNA datasets, rRNA and YRNA datasets, genomic tRNA datasets, mitochondrial tRNA datasets, piRNA datasets, and non-coding RNA datasets to count the read numbers of sncRNA transcripts using Bowtie 25.

### Detection of differentially expressed sncRNAs

Differentially expressed sncRNAs were identified using the R package edgeR (v3.36.0) 26. A sncRNA was considered to be significantly differentially expressed at *p* <= 0.05 and the absolute value of log_2_-fold change >= 1.

### Validation of sncRNA expression by RT-PCR, quantitative RT-PCR and northern blot

We used reverse transcription to validate sncRNAs as described 13. the sncRNA primers used are displayed in **Table S2**. Similar volumes of serum and spike-in control (cel-miR-39) were used to normalize samples. Northern blotting was performed to verify the expression pattern of tsRNA-Gly^CCC^, ysRNA^RNA4^, and ysRNA^RNY5^ in serum from healthy controls, as described 27. Briefly, total RNA from 1 ml serum was electrophoresed by 15% urea-PAGE gel, and transferred to a Nytran Super Charged membranes (Roche, Basel, Switzerland) followed by UV cross-linking. The membranes were incubated with DIG-labelled oligonucleotide probes in hybridization solution (Roche) overnight (12-16 h) at 42 °C. DIG-labelled oligonucleotide probes were synthesized by Takara (Iwate, Japan), and the sequences are displayed in **Table S3**.

### Machine learning marker sncRNA discovery and performance evaluation

We used Random Forest (v4.6-14) 28 in R to evaluate binary classifiers based on several small noncoding RNA datasets. Our strategy for sncRNA signature selection was based on the following: (1) evidence of differential expression; (2) average expression level > 10; and (3) significance (p < 0.05) in logistic regression.

### Statistical analyses

Statistical significance was determined using Student's t test and one- and 2-way ANOVAs with Fisher's LSD test. Pairwise correlations between blood and bone marrow sncRNA expression profiles were calculated by Pearson correlation analyses.

### Data sharing statement

Part of the sncRNA sequencing datasets is available in the Genome Sequence Archive for Human database (GSA-Human, Beijing Institute of Genomics, Chinese Academy of Sciences) under the accession number HRA001179.

Complete methods are included in the **Supplementary Materials**.

## Results

### Over 20 types of sncRNAs beyond miRNAs were identified in human serum

**Figure 1:**
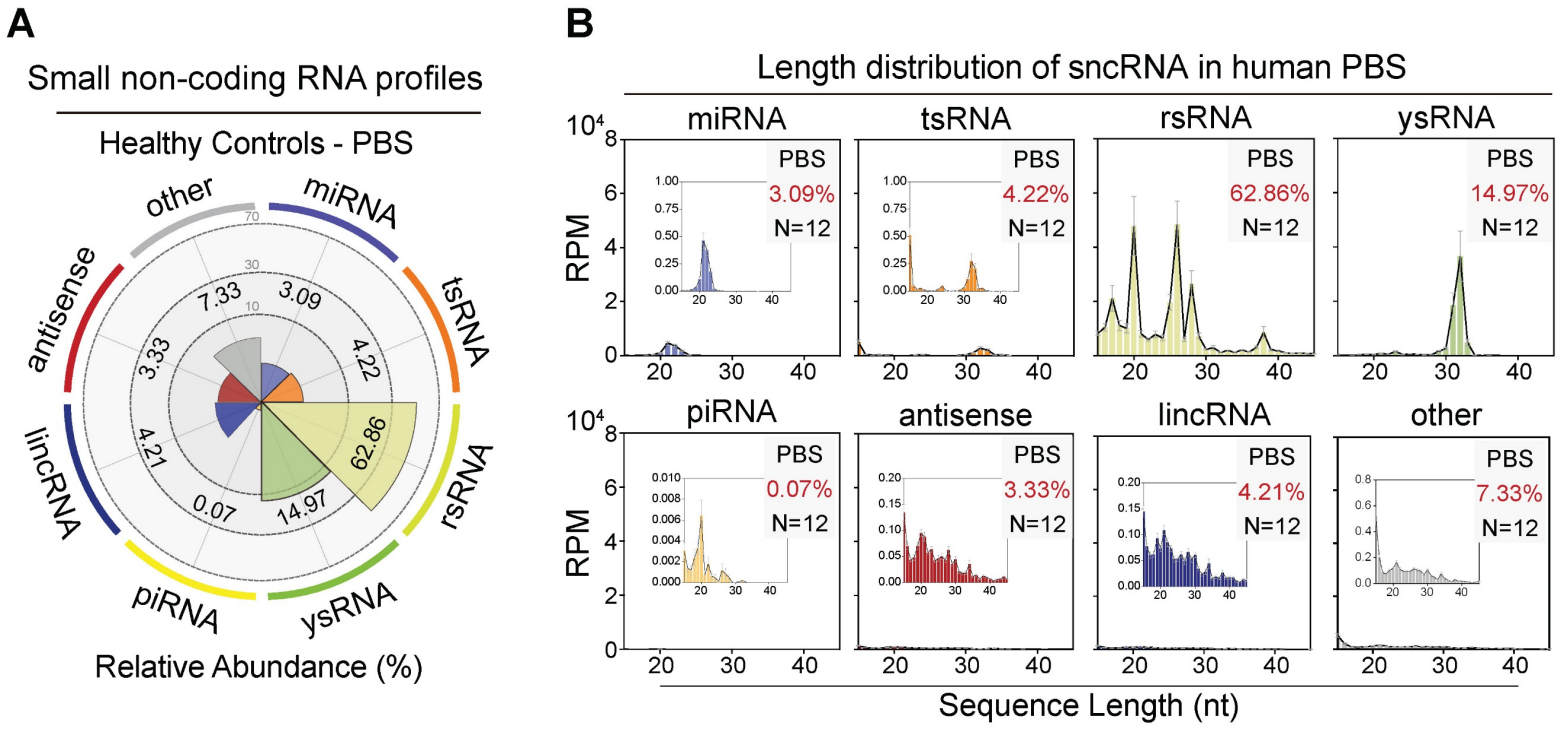
** The small non-coding RNA landscape in healthy human peripheral blood serum (PBS).** (A) Relative proportion of seven major sncRNA categories in healthy individuals (n = 12). (B) Length distribution of selected sncRNA types in healthy individuals (n = 12). The 'other' group includes sncRNAs derived from miscellaneous RNAs, snoRNAs, snRNAs, processed transcript, etc. The X-axis represents the nucleotide length (nt) and the Y-axis represents the expression level (RPM, reads of exon model per million mapped reads) of each type of sncRNA.

To overcome the limitation that exists in the previous miRNA-biased sncRNA detection methods and enrich small RNA categories in human serum, we performed small RNA sequencing with a 15-45 nt nucleotide length range to characterize the sncRNA landscape in the human haematological system. In our study**,** over 17% of total miRNAs (237/1,373) were detected in serum, and 102 miRNAs showed an expression level > 10 (**Figure S1A-C**). Using the small non-coding RNA analysis software SPORTS, we identified over 20 types of sncRNA categories beyond miRNAs, including tsRNAs, rsRNAs, ysRNAs, piRNAs, anti-senses, lincRNAs, snRNAs, and snoRNAs (**Figure 1A-B and Figure S2A**) 24. Interestingly, the most dominant small RNAs among circulating RNAs were not well-known miRNAs but a class of rRNA-derived small non-coding RNAs, accounting for an average of 62.86% (**Figure 1A**). tsRNA was previously reported to be enriched in vertebrate serum 13. In the present study, we identified a comparable proportion of tsRNA with miRNA in human serum (3.09% miRNA vs. 4.22% tsRNA). Moreover, an appreciable amount of ysRNA, accounting for an average of 14.97%, was identified. In addition, the levels of rsRNA, tsRNA, and ysRNA constituted over 80% of the total circulating sncRNA, with piRNAs and other sncRNAs comprising the remaining 15% (**Figure S2A**).

### rsRNAs and ysRNAs are dominant in human PBS with distinct distribution features

**Figure 2:**
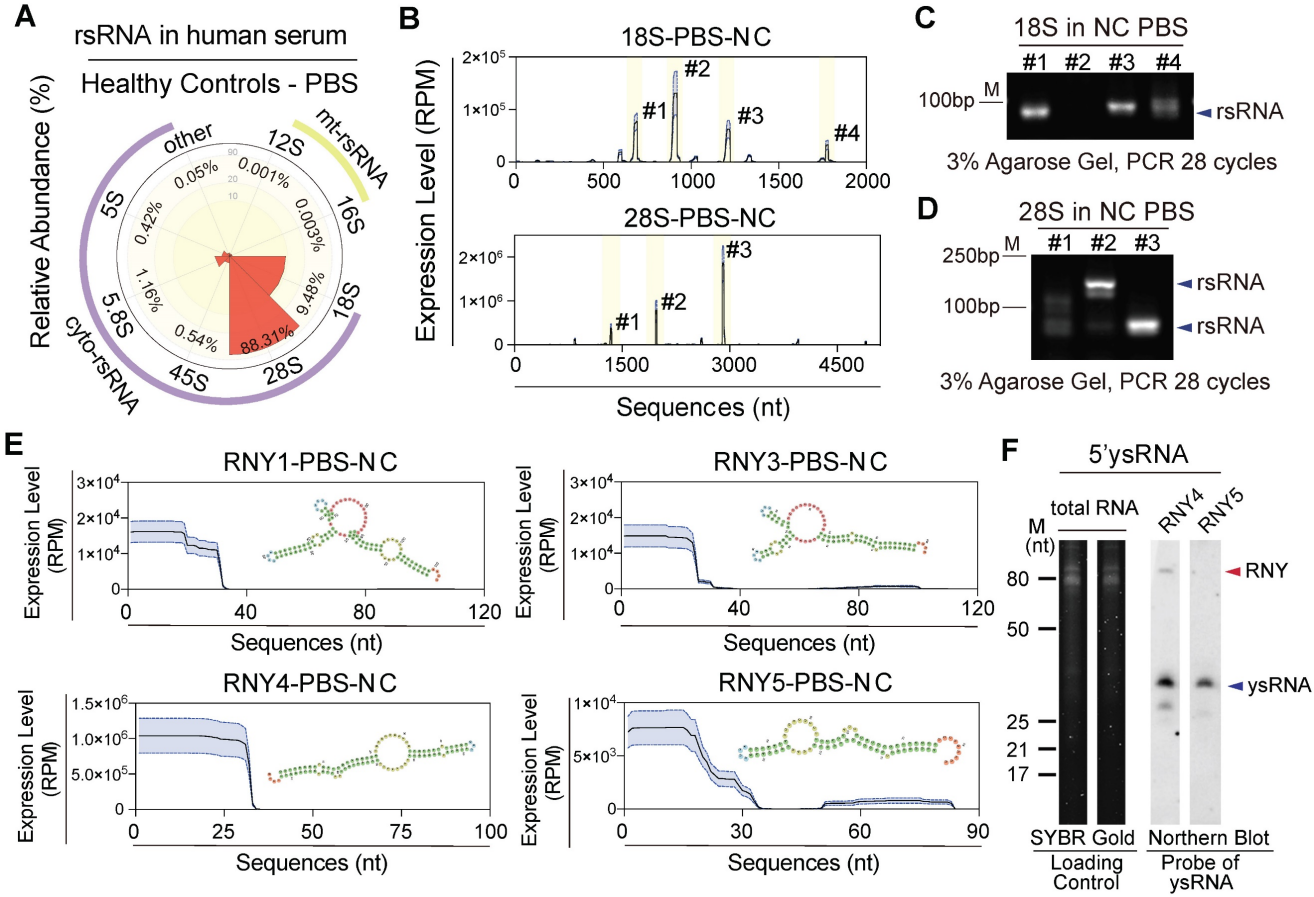
** Characterization of rsRNA- and ysRNA-generating loci in healthy human PBS.** (A) Distribution of five nucleus-encoded rsRNAs (cyto-rsRNAs: 5S, 5.8S, 18S, 28S, and 45S) and two mitochondria-encoded rsRNA (mt-rsRNAs: 12S and 16S) categories. (B) Expression peaks and localization of cyto-rsRNA-18S and 28S. Expression levels are presented as mean ± SEM. (C-D) Validation of selected rsRNA main peaks by RT-PCR, (C) rsRNA-18S peaks, and (D) rsRNA-28S peaks; blue arrowheads indicate rsRNAs. (E) Structure schematic and nucleotide mapping of ysRNA^RNY1^, ysRNA^RNY3^, ysRNA^RNY4^, and ysRNA^RNY5^. Expression levels are presented as mean ± SEM. (F) Northern blot validation of 5'ysRNA^RNY4^ and 5'ysRNA^RNY5^ in human PBS, at least three healthy individual PBS samples were used for validation.

rsRNAs have been ignored as rRNA debris for decades due to the large amount of rRNA in cells 29. However, Zhang et al. found that 28S rRNA-derived rsRNA was enriched in mouse mature sperm under physiological conditions and was increased in multiple tissues after LPS infection, which indicated the potential roles of rsRNAs involved in body active inflammation 13. Our study found that in addition to miRNAs, a large number of rsRNAs are present in human PBS (**Figure 1A**). By mapping and comparing the overall expression pattern of different rsRNA classes and sequence locations, we further classified rsRNAs into nucleus and mitochondria encoded (**Figure 2A**). Systematic analyses indicated that 28S-rsRNAs (88%) and 18S-rsRNAs (10%) constituted > 98% of the total rsRNAs (**Figure S3A-B**). We found specific sequence origination and distribution patterns for different rsRNAs (**Figure 2B and Figure S3C-D**). 18S-rsRNAs exhibited four main sequence peaks distributed in locations 674-692 (#1), 897-919 (#2), 1,194-1,223 (#3) and 1,838-1,862 (#4; **Figure 2B-C and Figure S3E**). 28S-rsRNAs had three main sequence peaks at locations 1,336-1,355 (#1), 1,963-1,982 (#2) and 2,895-2,920 (#3; **Figure 2B, D and Figure S3F**).

Human YRNAs are a group of small non-coding RNAs with nucleotide lengths ranging from 83-113 nt that were originally described in systemic lupus erythaematosus 30. There are four members in the human YRNA family (**Figure S4A**), namely, RNY1, RNY3, RNY4, and RNY5, which can be cleaved by RNsase1 to produce ysRNAs. Studies have shown that ysRNAs are abundant in extracellular spaces, such as in serum, plasma, and other biofluids, in humans 31, 32. In this study, we identified abundant ysRNAs mostly derived from the 5' end of YRNAs with nucleotide lengths of approximately 30 nt (**Figure 1B and 2E**). Composition analyses and northern blot validation indicated that ysRNA^RNY4^ concentrations were greater than those of ysRNA^RNY1^, ysRNA^RNY3,^ and ysRNA^RNY5^ (**Figure 2F and Figure S4B-C**).

### Comprehensive analysis of the expression patterns of tsRNAs in human serum

**Figure 3:**
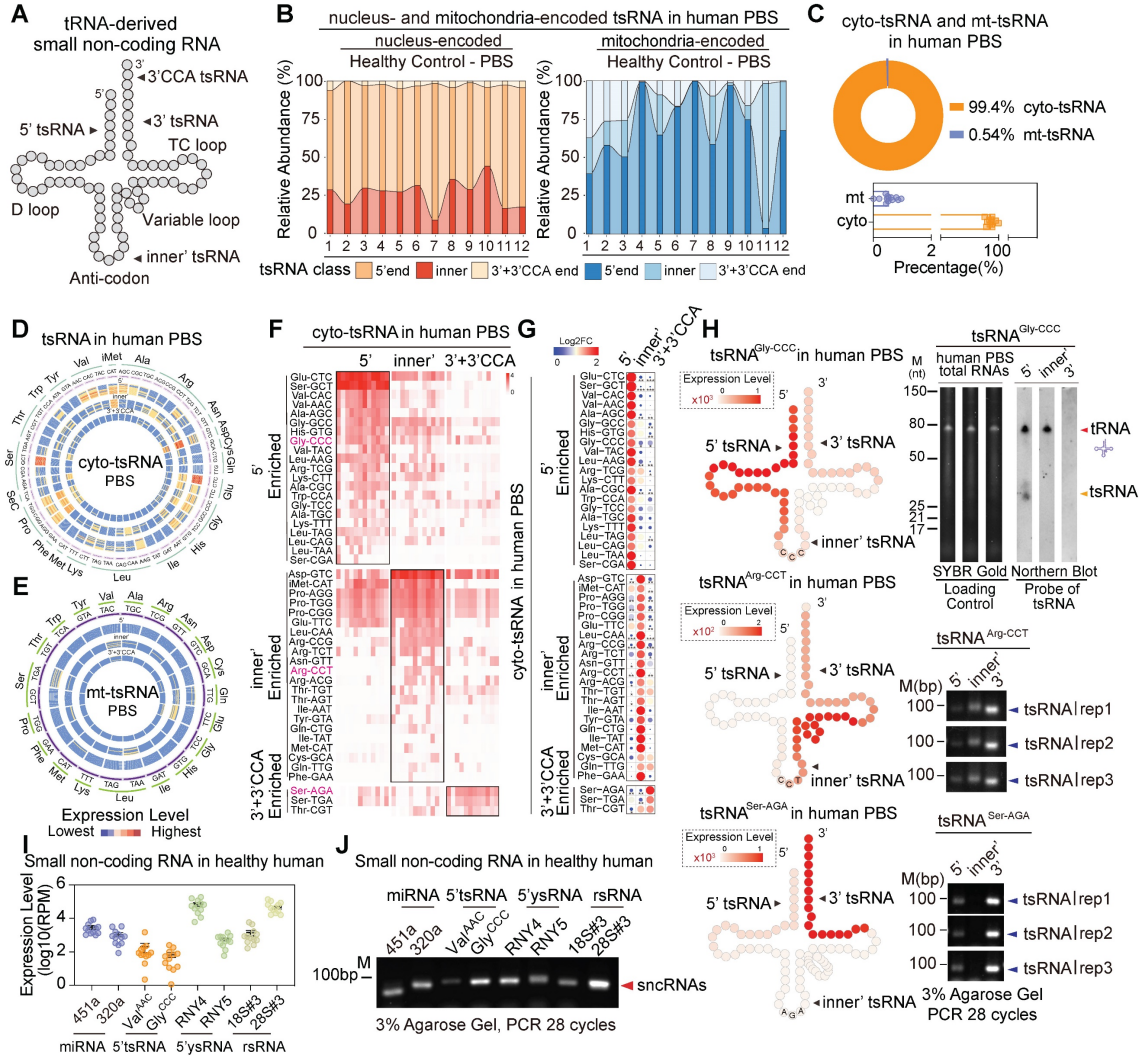
** Expression patterns of tsRNAs in healthy human PBS.** (A) Schematic illustration of the four distinct categories of tRNA-derived small non-coding RNAs (tsRNAs): 5'tsRNAs, inner'tsRNAs, 3'tsRNAs, and 3'CCA tsRNAs. (B) Relative abundances of three distinct tsRNA categories (5', inner', 3'+3'CCA) for cyto-tsRNAs and mt-tsRNAs. (C) Proportional distribution of cyto-tsRNAs and mt-tsRNAs in human PBS. (D-E) Cyto-tsRNA and mt-tRNA expression profiles are categorized by anti-codons of amino acids in healthy PBS, 5'tsRNAs, inner'tsRNAs, and 3'+3'CCA tsRNAs are shown from outer circles to inner circles, respectively. Each circle represents one sample. (F-G) The heatmap represents 5'enriched, inner'enriched, and 3'+3'CCA'enriched cyto-tsRNAs. (H) Schematic illustration of single-base resolution and validation for the origination of tsRNA^Gly-GCC^, tsRNA^Arg-CCT^, and tsRNA^Ser-AGA^ in human PBS by RT-PCR or northern blot, at least three healthy individual PBS samples were used for validation. (I) Expression level comparison of the top two expressed sncRNAs in each category of healthy human PBS. (J) Expression level comparison of the top two expressed sncRNAs in each category of human PBS by RT-PCR, at least three healthy individual PBS samples were used for validation.

tsRNAs, also known as tRFs, have been shown to play pivotal roles in cellular transcriptional and translational control in response to various cell stresses 4, 33, 34. Recently, tsRNAs have emerged as diagnostic biomarkers in cancers and are functionally involved in human physiological and pathological processes 35-38. In the study, the overall amount of tsRNAs identified in the human extracellular circulating system was comparable to that of miRNAs (**Figure 1A**). We grouped tsRNAs into three distinct categories according to sequence annotation and major cleavage sites on mature tRNAs, which are mainly driven by ANG, Dicer, RNaseZ, and RNaseP: 5'tsRNAs, inner'tsRNAs, and 3'tsRNAs (**Figure 3A**). Next, we combined 3'tsRNAs with or without a CCA tail. Our data indicate that genomic tRNA-derived tsRNAs (cyto-tsRNAs) were generated by cleavage of the 5' end of mature tRNA around the anticodon loop, namely, 5'tsRNAs (70% of total cyto-tsRNAs; **Figure 3B**), such as cyto-5'tsRNA^Glu^ and cyto-5'tsRNA^Ser^. However, unlike cyto-tsRNAs, mitochondrial tRNA-derived tsRNAs (mt-tsRNAs) were largely skewed towards generating inner'tsRNAs and only accounted for a small proportion of the total circulating tsRNAs (**Figure 3B-C**). We also found that different tRNAs, including cyto-tRNAs and mt-tRNAs, generated different subtypes of tsRNAs. (**Figure 3D-G and Figure S5A**-**B**). For example, cyto-tRNA^Gly^ generated 5'tsRNAs, whereas cyto-tRNA^Arg^, cyto-tRNA^Ser^, and cyto-tRNA^Thr^ generated inner'tsRNAs or 3'tsRNAs (**Figure 3F**-**G**). This tsRNA biogenesis preference was validated by northern blot and RT-PCR (**Figure 3H and Figure S5C**-**D**).

Considering that many biotypes of sncRNAs showed high expression levels in human serum, we randomly selected and compared relatively enriched sncRNAs in the group of miRNAs (mir-451a and mir-320a), tsRNAs (cyto-5'tsRNA^Val^ and cyto-5'tsRNA^Gly^), ysRNAs (5'ysRNA^RNY4^ and 5'ysRNA^RNY5^) and rsRNAs (rsRNA-18S#3 and rsRNA-28S#3). Notably, expression levels of rsRNAs, tsRNAs, and ysRNAs were comparable to those of the top two expressed miRNAs (**Figure 3I-J**). Their high enrichment in the human serum might also indicate pivotal roles in the human pathophysiological process.

### Circulating small RNAs differ widely between AML patients and controls

**Figure 4:**
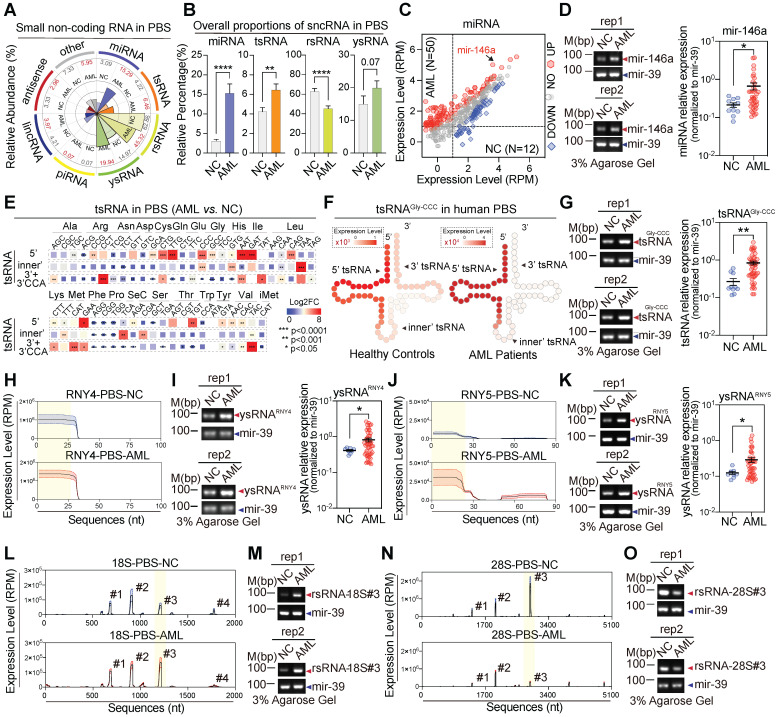
** SncRNA alteration signatures in AML patients.** (A) The comparative analysis illustrates the overall proportion of seven major sncRNA categories between healthy controls and AML patients. (B) The histogram shows the alteration of four sncRNAs including miRNAs, tsRNAs, rsRNAs, and ysRNAs between healthy controls and AML patients. Statistical significance for the analyses was determined by the t test (*p < 0.05, ***p < 0.001, ****p < 0.001, NS > 0.05), and error bars display the mean ± SEM. (C) Scattered plot comparison of profile changes in miRNAs in AML patients vs. healthy controls in PBS. (D) Validation of mir-146a by RT-PCR and quantitative RT-PCR. (E) Comparison of cyto-tsRNA expression profiles categorized by the anti-codons of amino acids between healthy controls and AML patients. Statistical significance for the analyses was determined by the t test (**p < 0.01, ***p < 0.001). (F-G) Schematic illustration for single-base resolution and RT-PCR and quantitative RT-PCR validation for the origination of tsRNA^Gly-GCC^ in healthy controls and AML patients. (H) Expression signature and sequence mapping location of ysRNA^RNY4^ in human PBS on their parental RNY4. Expression levels are presented as mean ± SEM. (I) Validation of 5'ysRNA^RNY4^ by RT-PCR and quantitative RT-PCR. (J) Expression signature and sequence mapping location of ysRNA^RNY5^ in human PBS on their parental RNY5. Expression levels are presented as mean ± SEM. (K) Validation of 5'ysRNA^RNY5^ by RT-PCR and quantitative RT-PCR. (L) Expression signature and sequence mapping location of rsRNA-18S in human PBS on their parental rRNA. Expression levels are presented as mean ± SEM. (M) Validation of rsRNA-18S (#3) by RT-PCR and quantitative RT-PCR. (N) Expression signature and sequence mapping location of rsRNA-28S in human PBS on their parental rRNA. Expression levels are presented as mean ± SEM. (O) Validation of rsRNA-28S (#3) by RT-PCR and quantitative RT-PCR.

To further understand the comprehensive expression profiles of human circulating sncRNAs and generate high confidence fingerprints of serum sncRNAs in AML patients, we enrolled 50 preliminarily diagnosed patients with de novo AML and collected the serum for sncRNA-sequencing (**Figure S6A**). Consistent with the circulating sncRNA profiles in healthy controls we described previously, over 20 types of sncRNA were enriched in samples from the AML patients compared with the controls, including rsRNAs, tsRNAs, ysRNAs, and miRNAs (**Figure S6B-C**). We found that 179 miRNAs (38%) were up-regulated and 63 miRNAs (13%) down-regulated (**Figure S4C-D**). In the tsRNA cohort, fragments aligned to a distinct location of tsRNAs were detected and compared between the controls and AML patients. In both cohorts, cyto-5'tsRNAs were the predominant class of tsRNAs (**Figure S7A**). However, alterations were detected across different amino acids, anti-codon, and iso-acceptors of tsRNAs in AML patients compared with the controls (**Figure 4E-G and Figure S7B-G**). Among cyto-tsRNAs, we observed a large increase in cyto-5'tsRNA^Gly^, cyto-inner'tsRNA^Leu^, cyto-5'tsRNA^Lys^, cyto-inner'tsRNA^Val^ and cyto-5'tsRNA^Cys^ in the AML patients compared with healthy controls (**Figure 4G and Figure S7C-F**). ysRNAs were also increased in AML patients compared with controls (**Figure 4A-B**). Up-regulation of serum ysRNAs in AML correlated with increases in ysRNA^RNY4^ and ysRNA^RNY5^ (**Figure 4H-K and Figure S8A-B**). Unlike other sncRNAs, there was a strong reduction in rsRNAs (**Figure 4A-B**). A slight proportional alteration was observed in the component of sub-rRNA classes (**Figure S9A-C**); for example, cyto-rsRNA-18S (#3) was significantly increased (**Figure 4L**-**M**), whereas cyto-rsRNA-28S (#3) was significantly decreased (**Figure 4N**-**O**). Such dynamic dysregulation of circulating sncRNAs in AML patients suggests potential diagnostic applications.

### tsRNA-based feature screening robustly discriminates subjects with AML from controls

**Figure 5:**
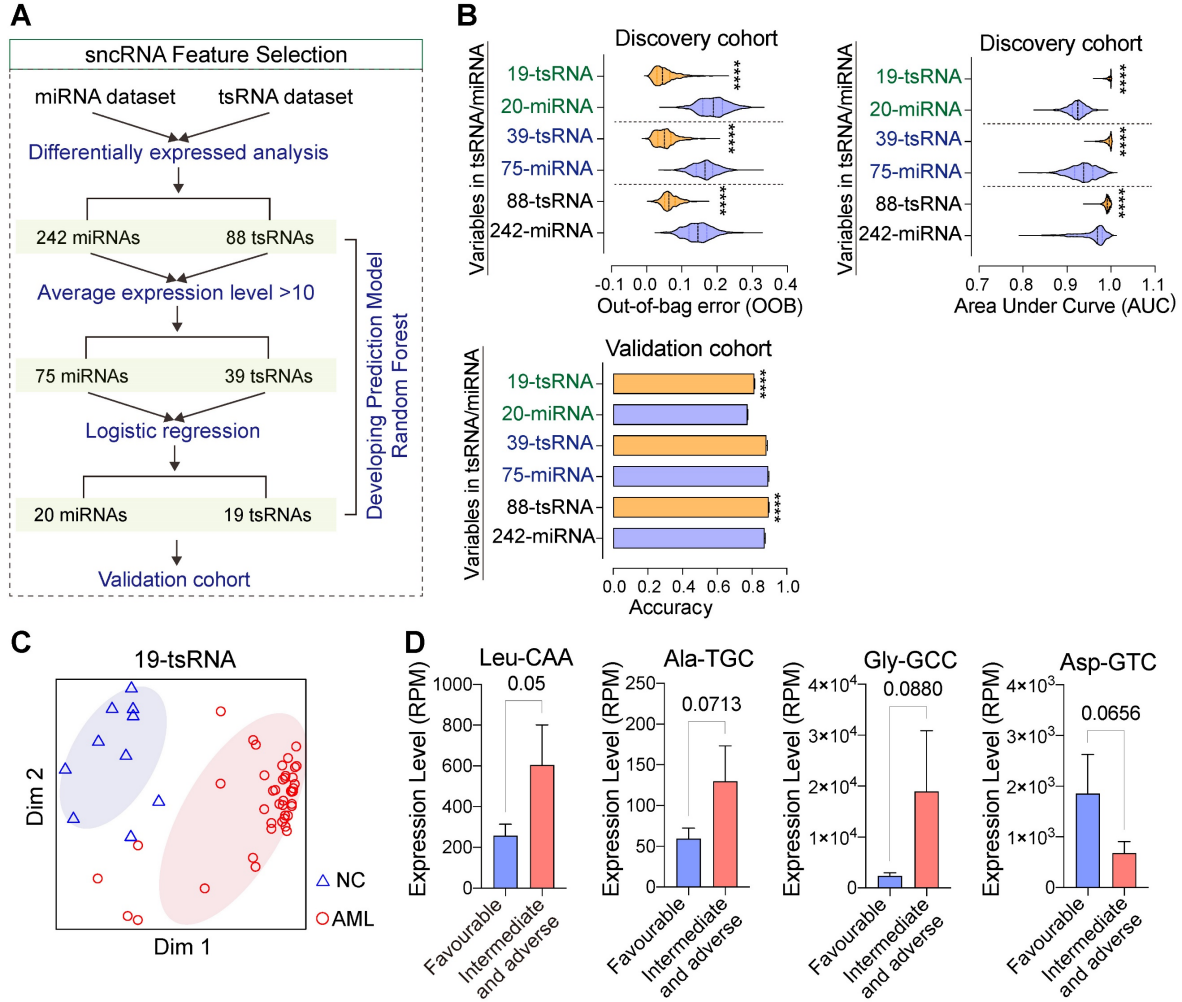
** tsRNA-based feature screening robustly discriminates subjects with AML from controls.** (A) Strategies and workflow of screening miRNA and tsRNA characteristics for developing the prediction model. (B-C) Performance of distinct miRNA and tsRNA datasets in the discovery and validation cohorts. AUC: area under the receiver operating characteristic curve, OOB: out of bag error. (D) Several tsRNAs at the time of diagnosed correlated with risk levels.

To evaluate the potential roles of circulating sncRNAs, we used machine learning methods to develop and evaluate binary classifiers (**Figure 5A**). Our data indicate that fewer tsRNA signatures performed better in discriminating persons with AML from controls compared with miRNAs in both discovery and validation cohorts (**Figure 5B**-**C and Figure S10A**). These findings demonstrate that tsRNA-based feature screening signatures were able to robustly discriminated individuals with AML from controls. AML patients are classified into adverse, intermediate, and favourable risk categories on the basis of cytogenetic and molecular abnormalities, and we found that levels of several sncRNAs (tsRNAs and miRNAs) at diagnosis correlated with risk level. For example, high levels of tsRNA^Ala-TGC^, tsRNA^Leu-CAA^, and tsRNA^Gly-GCC^ were associated with an intermediate or adverse prognosis but low levels of tsRNAs with a favourable prognosis, indicating that serum tsRNAs might also be closely associated with AML progression and prognosis (**Figure 5D and Figure S10B**).

### Coordinate sncRNA signatures between blood and bone marrow samples

**Figure 6:**
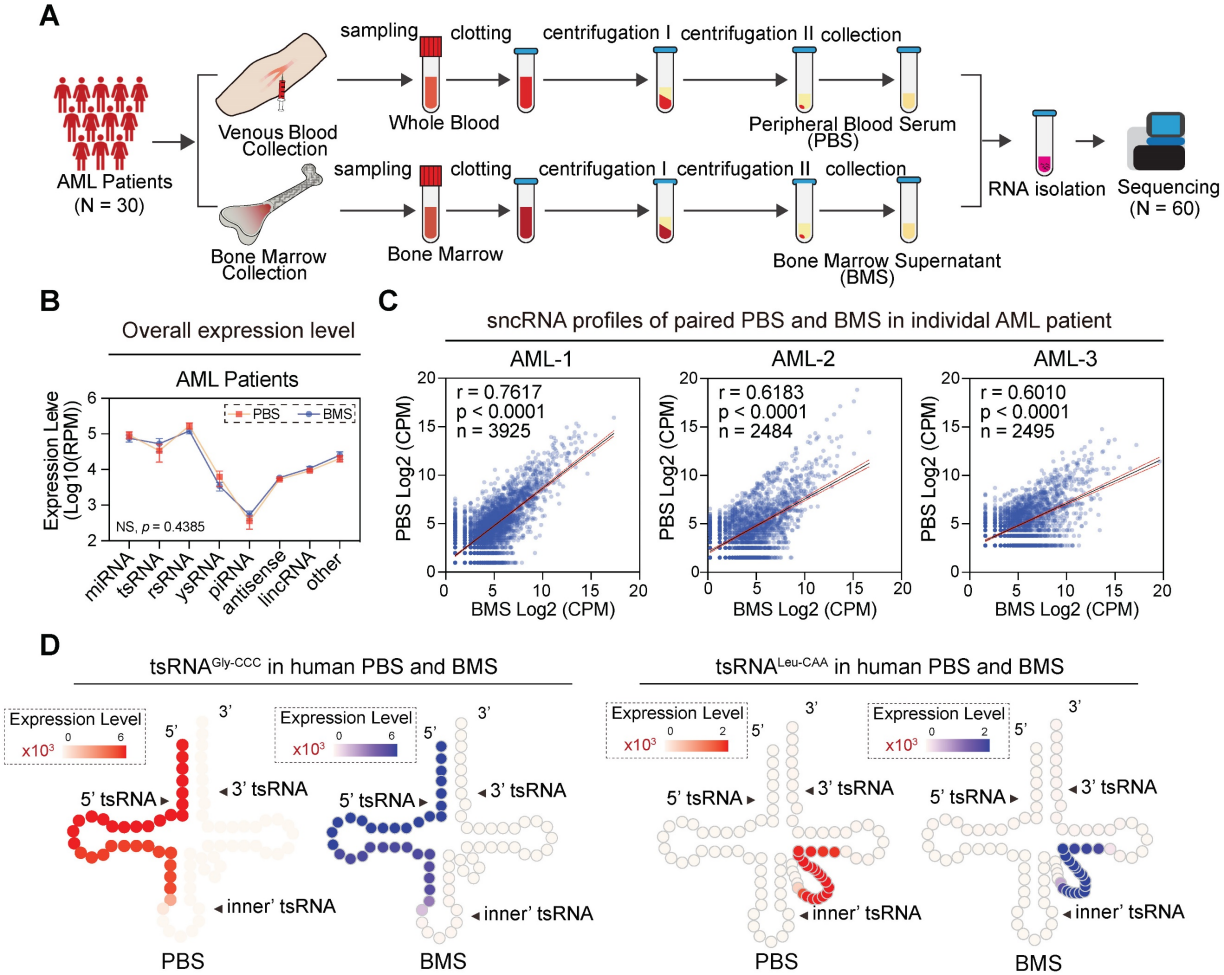
** Correlation analysis of sncRNA expression levels between PBS and BMS in AML patients.** (A) In the pairwise analysis cohort, 60 libraries including paired PBS and BMS were collected from 30 AML patients and profiled by sncRNA-seq. (B) Comparison of overall sncRNA expression levels in AML PBS and BMS. The expression level was measured with Log10 (RPM), and error bars display the mean ± SEM. Statistical significance was determined by two-way ANOVA with the Fisher's LSD test (NS > 0.05). (C) Scatter plot showing the correlation of sncRNA expression profiles between paired PBS and BMS samples derived from an individual in the AML group. (D) Schematic illustration for single-base resolution for the origination of tsRNA^Gly-GCC^ and tsRNA^Leu-CAA^ in PBS and BMS of AML patients.

Newly-diagnosed individuals with AML generally showed similar flow cytometric and cytogenetic analysis results between blood and bone marrow 39. To investigate whether the sncRNA profile was similar in those samples, we collected paired samples from 30 newly-diagnosed subjects and generated 60 paired sncRNA-seq datasets (**Figure 6A**). Interestingly, the sncRNA profiles in peripheral blood serum (PBS) and bone marrow supernatants (BMS) from individual AML patients seemed to be remarkably consistent. No significant difference was observed in overall expression levels of various types of sncRNAs among blood and bone marrow derived from individual AML patients (**Figure 6B-C**). Moreover, highly consistent expression patterns of individual sncRNAs were obtained from various types of sncRNAs, especially miRNAs, tsRNAs, rsRNAs, and ysRNAs (**Figure S11**). Importantly, expression patterns of 6-tsRNAs capable of distinguishing AML patients from healthy controls showed remarkable consistency between PBS and BMS, such as tsRNA^Ala-TGC^, tsRNA^Gly-CCC^, tsRNA^Leu-CAG^, tsRNA^Leu-CAA^ and tsRNA^Lys-TTT^ (**Figure 6D**). These results demonstrated that PBS and BMS from AML patients shared highly consistent sncRNA signatures and suggest that the circulating sncRNA in PBS and BMS might contribute to shaping the microenvironment of leukaemia for leukaemogenesis.

## Discussion

AML is a highly heterogeneous disorder at the molecular and clinical levels that is generally caused by gradually accumulating mutations, numerous additional genetic and epigenetic abnormalities, and post-transcription and translation regulator alterations 40-45. Due to their broad range of functions in post-translational regulation and the haematopoietic microenvironment, sncRNAs, especially miRNAs, play major roles in maintaining haematopoietic stem cell homeostasis and are well-documented in leukaemia progression, metastasis, and drug resistance 46-49. Many studies over the past decades have identified non-canonical sncRNAs such as tsRNAs, rsRNAs, and ysRNAs and revealed their versatile roles in cell homeostasis and disease 35, 50-55. To our knowledge, this study is the first to comprehensively characterize the sncRNA profiles of human blood serum and bone marrow supernatant from people with and without AML, revealing a specific sncRNA profile in AML. Many early studies focusing on sncRNAs aimed to discover miRNAs (21-23 nt) and siRNAs (20-27 nt) of ~20 nt using a pre-size selection of < 30 nt RNA 56, which prevented the discovery of sncRNAs > 30 nt, such as piRNAs (21-35 nt) and tsRNAs (30-40 nt). In this study, an RNA size in the range of 15-45 nt was selected for sequencing, which can cover many sncRNAs. It is encouraging that over 20 biotypes of sncRNAs are present in both blood and bone marrow. Furthermore, by describing their expression level, characteristic distribution, and source certification, we showed that rsRNAs are the most abundant sncRNAs in human serum and are mainly derived from 18S- and 28S-rRNAs. We also characterized the expression pattern of tsRNA and ysRNA, which are mainly derived from the 5' end of their parental RNAs, in human serum. This is in agreement with other reports that 5'tsRNAs are ubiquitously expressed and relatively stable in cells 57. Such a complete landscape of sncRNAs suggests that in additional to miRNAs, other serum-enriched sncRNAs (e.g., tsRNAs, rsRNAs and ysRNAs) might also be involved in the onset, development, and pathogenesis of leukaemia. In general, our work provides an exhaustive list of sncRNAs in the blood and bone marrow of healthy participants and AML patients, which is a prerequisite for basic elucidation of their functions.

By screening the sncRNA transcriptome in healthy participants and AML patients and validating it by multiple technologies (qPCR, RT-qPCR and northern blot), we found that the category and origin of most serum sncRNAs did not change significantly. Serum tsRNAs and ysRNAs presented a uniform length distribution and 5' end dominance, indicating conserved biological processes. However, significant expression alterations were detected in AML patients across various sncRNAs, such as cyto-5'tsRNA^Gly^, cyto-inner'tsRNA^Leu^, cyto-5'tsRNA^Lys^, cyto-5'tsRNA^Cys^, ysRNA^RNY4^, and ysRNA^RNY5^, indicating that serum sncRNAs have experienced reshaping due to the activities of cleavage enzymes (e.g., ANG, Dicer, RNaseZ, and RNaseP) and the modifications they harbor (e.g., m7G, m6A, m5C). Increasing evidence has shown that post-transcriptional modification under the catalysis of different modifying enzymes is necessary and affects all aspects of tRNA biology 58. Abnormal modification states can both affect codon decoding and impact tsRNA generation, which is sufficient to promote cancer initiation 37, 59. Therefore, our study also indirectly presents that epitranscriptomics changes occur in AML patients, which might be the consequence of the high proliferative and metabolic state of cancer cells.

In general, tsRNA and rsRNA are characterized by multiple RNA modifications and non-standard 3' and 5' termini when compared to miRNA (3'-hydroxyl and 5' -phosphate), which would block the adaptor ligation process and interfere with reverse transcription during cDNA library construction. To overcome these problems, we and others recently established a novel tsRNA- and rsRNA- friendly sncRNA sequencing method (PNDORA-Seq 60, AQRNA-Seq 61, and CPA-Seq 62), which expands our knowledge on the repertoire of sncRNAs in mouse tissues and human ES cells. Nevertheless, application of these methods requires a large amount of total RNA input, which cannot be obtained from human serum, especially from AML patients. We believe that the development of these methods with trace RNA input will allow for an informative repertoire of sncRNAs in the human haematological system and improve clinical diagnosis.

Recently, sncRNAs have been implicated in multiple diseases, and their dysregulation is involved in regulating leukaemia cell growth, metastasis, and communication through multiple pathways 37, 59, 63. Although most of the functions of sncRNAs are still poorly understood, their capabilities as biomarkers for disease diagnosis and prognosis have been revealed in clinical studies 15, 36. In our study, by applying highly efficient machine learning methods for classification, we found that certain sncRNA combinations (such as tsRNAs and miRNAs) could effectively distinguish healthy participants from AML patients, with fewer tsRNAs performing better, suggesting that it may be worthwhile to investigate whether any sncRNA candidates have functional roles that affect leukaemia growth. Moreover, our results showed that tsRNAs are present at a considerably or even higher level than miRNAs in human serum, which is conducive to their use as a measurable biomarker. In addition to miRNAs and tsRNAs, ysRNAs and rsRNAs show significant dysregulation in overall proportions and displayed significant up-regulation or down-regulation. However, we did not use ysRNAs and rsRNAs for modelling in our study for two reasons. 1) They have a relatively narrow range of features. By generating multiple alignments, we found that dominantly expressed ysRNAs - 5'ysRNA, especially produced by RNY1, RNY3, and RNY4, shared over 70% nucleotides. Although those sequences can be easily separated by sncRNA-seq, they are difficult to distinguish in the clinical by qRT-PCR, RT‒PCR, or northern blotting during detection. 2) They have complicated distribution features. Unlike miRNAs, tsRNAs, or ysRNAs, rsRNAs usually generate more than one main peak (such as 28S-rRNAs or 18S-rRNAs), and each rsRNA peak has a wide sequence length (15-45 nt), which makes it difficult to precisely locate and quantify levels of target rsRNAs, especially in clinical applications.

Early assessment of patient prognosis can dramatically improve chances of survival. Specific sncRNAs in the serum of those with solid tumours have great value for pathologic diagnosis, assessment of malignant grade, evaluation of curative effect, and prognosis prediction of malignancy 64, 65. Our results for AML patients showed that expression levels of some tsRNA at diagnosis correlated with risk, which may be valuable to encourage further studies on the specific contributions of tsRNA to AML biology. We further analysed the correlation between levels of tsRNA and clinical and biological characteristics, such as the number of myeloblasts. Unfortunately, no significant correlations were found, indicating that the sncRNAs discovered in this study may also be produced by alterations in physiologic changes in inflammation/metabolism, by the tumour microenvironment, or as a result of leukaemia growth. In addition, our sample size was small (n = 122), which limited our ability to better clarify the correlation between tsRNA level and clinical characteristics, which will be further explored in our next project by expanding samples, as will the specific function of tsRNA in AML.

Haematological malignancy is often located and develops in the bone marrow where blood is produced, followed by uncontrolled growth of abnormal blood cells, interfering with regular functions of normal blood cells and breakage of the marrow blood barrier and leading to immature blood cells leaving the bone marrow to the peripheral blood. In the clinical, bone marrow aspiration and biopsy are commonly used as the recommended diagnostic approach for haematological malignancies, especially AML, despite their invasiveness. Previous studies have shown considerable concordance between the blood and bone marrow of newly diagnosed AML patients in terms of morphologic features, cytochemistry, immunophenotype, and leukaemia marker expression 39, 66. Therefore, it will be more convenient to use peripheral blood for studies if it can serve as well as bone marrow. In our study, sncRNA profiling of paired primary AML blood and bone marrow samples enabled the discovery of similarity and diversity. Through comprehensive analysis of the present data, we revealed that the majority of sncRNA compounds detected (including the type of compounds and the expression level) in bone marrow were also detected in blood. Substantial proportions of sncRNAs demonstrated moderate or strong correlations in blood and bone marrow (0.80 for miRNA, 0.67 for tsRNA, 0.90 for ysRNA, and 0.66 for rsRNA). However, the abundances of some specific sncRNAs, antisense RNA, and lincRNA, did not correlate significantly. This result suggests that the distinct biogenesis or function of antisense RNAs and lincRNAs is different from that of sncRNAs or that the profiles of antisense RNAs and lincRNAs might be tissue-specific, which may be associated with bone marrow microenvironment and has a key role in regulating haematopoiesis. Overall, our findings have wide implications and are appropriate for estimating and investigating bone marrow metabolism and tumour microenvironment studies in AML because the sncRNA footprints in blood showed a positive correlation with bone marrow and can be collected in a non-invasive way.

However, limited by its retrospective clinical research nature, our study still had shortcomings and should be interpreted with caution. First, to obtain a high-quality sequencing dataset, during the period the samples were taken, we discarded a certain amount of low-quality samples. Limited number of samples restricted our ability to perform analyses stratified by clinical characteristics. In addition, although we used cross-validation and independent cohorts, our models still need to be validated in a larger independent cohort. Another limitation is that as controls, we used blood from healthy participants. It can be argued that it is inappropriate to perform comparative analysis of predominately leukaemia myeloblasts with those containing less than 5% normal myeloblasts. To address this issue, adding a group such as persons with infection and other non-AML causes in future studies will be appropriate.

## Conclusions

This study provides a comprehensive characterization of human circulating sncRNAs and their alteration signature among healthy controls and AML patients, representing a unique resource for future biological studies that may help in identifying novel critical sncRNAs involved in leukaemia biology, prognosis, and drug resistance. Furthermore, we generated a serum tsRNA subset that showed robust performance and accuracy in distinguishing AML patients from healthy controls by machine learning methods, indicating the potential application of human serum tsRNAs as non-invasive biomarkers in AML diagnosis. Moreover, we observed closely coordinated regulation patterns of sncRNAs between blood and bone marrow from AML patients, shedding new light on employing blood sncRNA analysis in AML patients as a complementary diagnostic method for bone marrow aspiration and biopsy in assistance of AML diagnosis.

## Supplementary Material

Supplementary figures and tables, methods.

## Abbreviations

AML: acute myeloid leukaemia
AUC: area under the receiver operating characteristic curve
BMS: bone marrow supernatants
FAB classification: french-american-british classification
lincRNA: long intergenic non coding RNA
miRNA: microRNA
misc_RNA: miscellaneous RNA
mt-rsRNA: mitochondria-encoded rRNA-derived small RNA
mt-tsRNA: mitochondria-encoded tRNA-derived small RNA
nt: nucleotide length
PBS: peripheral blood serum
piRNA: piwi-interacting RNA
rsRNA: rRNA-derived small RNA
RT-PCR: reverse transcription-polymerase chain reaction
scaRNA: small cajal body-specific RNA
sncRNA: small non-coding RNA
snoRNA: small nucleolar RNA
snRNA: small nuclear RNA
tRNA: transfer RNA
tsRNA: tRNA-derived small RNA
ysRNA: YRNA-derived small RNA

## References

[1] Almstrup K, Lobo J, Morup N, Belge G, Rajpert-De Meyts E, Looijenga LHJ (2020). Application of miRNAs in the diagnosis and monitoring of testicular germ cell tumours. Nat Rev Urol.

[2] Mori MA, Ludwig RG, Garcia-Martin R, Brandao BB, Kahn CR (2019). Extracellular miRNAs: From Biomarkers to Mediators of Physiology and Disease. Cell Metab.

[3] Zhang B, Chen Z, Tao B, Yi C, Lin Z, Li Y (2021). m(6)A target microRNAs in serum for cancer detection. Mol Cancer.

[4] Shi J, Zhang Y, Zhou T, Chen Q (2019). tsRNAs: The Swiss Army Knife for Translational Regulation. Trends Biochem Sci.

[5] Shi J, Zhou T, Chen Q (2022). Exploring the expanding universe of small RNAs. Nat Cell Biol.

[6] Chu C, Yu L, Wu B, Ma L, Gou LT, He M (2017). A sequence of 28S rRNA-derived small RNAs is enriched in mature sperm and various somatic tissues and possibly associates with inflammation. J Mol Cell Biol.

[7] Gou LT, Dai P, Liu MF (2014). Small noncoding RNAs and male infertility. Wiley Interdiscip Rev RNA.

[8] Bartel DP (2018). Metazoan MicroRNAs. Cell.

[9] Ozata DM, Gainetdinov I, Zoch A, O'Carroll D, Zamore PD (2019). PIWI-interacting RNAs: small RNAs with big functions. Nat Rev Genet.

[10] Asano N, Matsuzaki J, Ichikawa M, Kawauchi J, Takizawa S, Aoki Y (2019). A serum microRNA classifier for the diagnosis of sarcomas of various histological subtypes. Nat Commun.

[11] Johnson P, Zhou Q, Dao DY, Lo YMD (2022). Circulating biomarkers in the diagnosis and management of hepatocellular carcinoma. Nat Rev Gastroenterol Hepatol.

[12] Valihrach L, Androvic P, Kubista M (2020). Circulating miRNA analysis for cancer diagnostics and therapy. Mol Aspects Med.

[13] Zhang Y, Zhang Y, Shi J, Zhang H, Cao Z, Gao X (2014). Identification and characterization of an ancient class of small RNAs enriched in serum associating with active infection. J Mol Cell Biol.

[14] Benard B, Gentles AJ, Kohnke T, Majeti R, Thomas D (2019). Data mining for mutation-specific targets in acute myeloid leukemia. Leukemia.

[15] Trino S, Lamorte D, Caivano A, Laurenzana I, Tagliaferri D, Falco G (2018). MicroRNAs as New Biomarkers for Diagnosis and Prognosis, and as Potential Therapeutic Targets in Acute Myeloid Leukemia. Int J Mol Sci.

[16] Stavast CJ, van Zuijen I, Karkoulia E, Ozcelik A, van Hoven-Beijen A, Leon LG (2022). The tumor suppressor MIR139 is silenced by POLR2M to promote AML oncogenesis. Leukemia.

[17] Liu Y, Cheng Z, Pang Y, Cui L, Qian T, Quan L (2019). Role of microRNAs, circRNAs and long noncoding RNAs in acute myeloid leukemia. J Hematol Oncol.

[18] Tian XP, Huang WJ, Huang HQ, Liu YH, Wang L, Zhang X (2019). Prognostic and predictive value of a microRNA signature in adults with T-cell lymphoblastic lymphoma. Leukemia.

[19] Reese M, Dhayat SA (2021). Small extracellular vesicle non-coding RNAs in pancreatic cancer: molecular mechanisms and clinical implications. J Hematol Oncol.

[20] Shiino S, Matsuzaki J, Shimomura A, Kawauchi J, Takizawa S, Sakamoto H (2019). Serum miRNA-based Prediction of Axillary Lymph Node Metastasis in Breast Cancer. Clin Cancer Res.

[21] Lee YR, Kim G, Tak WY, Jang SY, Kweon YO, Park JG (2019). Circulating exosomal noncoding RNAs as prognostic biomarkers in human hepatocellular carcinoma. Int J Cancer.

[22] Abe S, Matsuzaki J, Sudo K, Oda I, Katai H, Kato K (2021). A novel combination of serum microRNAs for the detection of early gastric cancer. Gastric Cancer.

[23] Wada Y, Shimada M, Murano T, Takamaru H, Morine Y, Ikemoto T (2021). A Liquid Biopsy Assay for Noninvasive Identification of Lymph Node Metastases in T1 Colorectal Cancer. Gastroenterology.

[24] Shi J, Ko EA, Sanders KM, Chen Q, Zhou T (2018). SPORTS1.0: A Tool for Annotating and Profiling Non-coding RNAs Optimized for rRNA- and tRNA-derived Small RNAs. Genomics Proteomics Bioinformatics.

[25] Langmead B, Trapnell C, Pop M, Salzberg SL (2009). Ultrafast and memory-efficient alignment of short DNA sequences to the human genome. Genome Biol.

[26] Robinson MD, McCarthy DJ, Smyth GK (2010). edgeR: a Bioconductor package for differential expression analysis of digital gene expression data. Bioinformatics.

[27] Zhang Y, Zhang X, Shi J, Tuorto F, Li X, Liu Y (2018). Dnmt2 mediates intergenerational transmission of paternally acquired metabolic disorders through sperm small non-coding RNAs. Nat Cell Biol.

[28] Breiman L (2001). Random Forests. Machine Learning.

[29] t Hoen PA, Friedlander MR, Almlof J, Sammeth M, Pulyakhina I, Anvar SY (2013). Reproducibility of high-throughput mRNA and small RNA sequencing across laboratories. Nat Biotechnol.

[30] Lerner MR, Boyle JA, Hardin JA, Steitz JA (1981). Two novel classes of small ribonucleoproteins detected by antibodies associated with lupus erythematosus. Science.

[31] Kowalski MP, Krude T (2015). Functional roles of non-coding Y RNAs. Int J Biochem Cell Biol.

[32] Nechooshtan G, Yunusov D, Chang K, Gingeras TR (2020). Processing by RNase 1 forms tRNA halves and distinct Y RNA fragments in the extracellular environment. Nucleic Acids Res.

[33] Chen Q, Zhang X, Shi J, Yan M, Zhou T (2021). Origins and evolving functionalities of tRNA-derived small RNAs. Trends Biochem Sci.

[34] Li J, Zhu L, Cheng J, Peng Y (2021). Transfer RNA-derived small RNA: A rising star in oncology. Semin Cancer Biol.

[35] Jin F, Yang L, Wang W, Yuan N, Zhan S, Yang P (2021). A novel class of tsRNA signatures as biomarkers for diagnosis and prognosis of pancreatic cancer. Mol Cancer.

[36] Zhu L, Li J, Gong Y, Wu Q, Tan S, Sun D (2019). Exosomal tRNA-derived small RNA as a promising biomarker for cancer diagnosis. Mol Cancer.

[37] Guzzi N, Muthukumar S, Ciesla M, Todisco G, Ngoc PCT, Madej M (2022). Pseudouridine-modified tRNA fragments repress aberrant protein synthesis and predict leukaemic progression in myelodysplastic syndrome. Nat Cell Biol.

[38] Fagan SG, Helm M, Prehn JHM (2021). tRNA-derived fragments: A new class of non-coding RNA with key roles in nervous system function and dysfunction. Prog Neurobiol.

[39] Weinkauff R, Estey EH, Starostik P, Hayes K, Huh YO, Hirsch-Ginsberg C (1999). Use of peripheral blood blasts vs bone marrow blasts for diagnosis of acute leukemia. Am J Clin Pathol.

[40] Welch JS, Ley TJ, Link DC, Miller CA, Larson DE, Koboldt DC (2012). The origin and evolution of mutations in acute myeloid leukemia. Cell.

[41] Trempenau ML, Schuster MB, Pundhir S, Pereira MA, Kalvisa A, Tapia M (2023). The histone demethylase KDM5C functions as a tumor suppressor in AML by repression of bivalently marked immature genes. Leukemia.

[42] Li Y, Wang C, Gao H, Gu J, Zhang Y, Zhang Y (2022). KDM4 inhibitor SD49-7 attenuates leukemia stem cell via KDM4A/MDM2/p21(CIP1) axis. Theranostics.

[43] Cuttano R, Colangelo T, Guarize J, Dama E, Cocomazzi MP, Mazzarelli F (2022). miRNome profiling of lung cancer metastases revealed a key role for miRNA-PD-L1 axis in the modulation of chemotherapy response. J Hematol Oncol.

[44] Ye S, Xiong F, He X, Yuan Y, Li D, Ye D (2023). DNA hypermethylation-induced miR-182 silence targets BCL2 and HOXA9 to facilitate the self-renewal of leukemia stem cell, accelerate acute myeloid leukemia progression, and determine the sensitivity of BCL2 inhibitor venetoclax. Theranostics.

[45] Zhang C, Shen L, Zhu Y, Xu R, Deng Z, Liu X (2021). KDM6A promotes imatinib resistance through YY1-mediated transcriptional upregulation of TRKA independently of its demethylase activity in chronic myelogenous leukemia. Theranostics.

[46] Wallace JA, O'Connell RM (2017). MicroRNAs and acute myeloid leukemia: therapeutic implications and emerging concepts. Blood.

[47] Schotte D, Pieters R, Den Boer ML (2012). MicroRNAs in acute leukemia: from biological players to clinical contributors. Leukemia.

[48] Peixoto da Silva S, Caires HR, Bergantim R, Guimaraes JE, Vasconcelos MH (2022). miRNAs mediated drug resistance in hematological malignancies. Semin Cancer Biol.

[49] Marcucci G, Mrozek K, Radmacher MD, Garzon R, Bloomfield CD (2011). The prognostic and functional role of microRNAs in acute myeloid leukemia. Blood.

[50] Kim HK, Fuchs G, Wang S, Wei W, Zhang Y, Park H (2017). A transfer-RNA-derived small RNA regulates ribosome biogenesis. Nature.

[51] Honda S, Loher P, Shigematsu M, Palazzo JP, Suzuki R, Imoto I (2015). Sex hormone-dependent tRNA halves enhance cell proliferation in breast and prostate cancers. Proc Natl Acad Sci U S A.

[52] Ivanov P, Emara MM, Villen J, Gygi SP, Anderson P (2011). Angiogenin-induced tRNA fragments inhibit translation initiation. Mol Cell.

[53] Goodarzi H, Liu X, Nguyen HC, Zhang S, Fish L, Tavazoie SF (2015). Endogenous tRNA-Derived Fragments Suppress Breast Cancer Progression via YBX1 Displacement. Cell.

[54] Lu S, Wei X, Tao L, Dong D, Hu W, Zhang Q (2022). A novel tRNA-derived fragment tRF-3022b modulates cell apoptosis and M2 macrophage polarization via binding to cytokines in colorectal cancer. J Hematol Oncol.

[55] Yang W, Gao K, Qian Y, Huang Y, Xiang Q, Chen C (2022). A novel tRNA-derived fragment AS-tDR-007333 promotes the malignancy of NSCLC via the HSPB1/MED29 and ELK4/MED29 axes. J Hematol Oncol.

[56] Carthew RW, Sontheimer EJ (2009). Origins and Mechanisms of miRNAs and siRNAs. Cell.

[57] Jehn J, Treml J, Wulsch S, Ottum B, Erb V, Hewel C (2020). 5' tRNA halves are highly expressed in the primate hippocampus and might sequence-specifically regulate gene expression. RNA.

[58] de Crecy-Lagard V, Boccaletto P, Mangleburg CG, Sharma P, Lowe TM, Leidel SA (2019). Matching tRNA modifications in humans to their known and predicted enzymes. Nucleic Acids Res.

[59] Orellana EA, Liu Q, Yankova E, Pirouz M, De Braekeleer E, Zhang W (2021). METTL1-mediated m(7)G modification of Arg-TCT tRNA drives oncogenic transformation. Mol Cell.

[60] Shi J, Zhang Y, Tan D, Zhang X, Yan M, Zhang Y (2021). PANDORA-seq expands the repertoire of regulatory small RNAs by overcoming RNA modifications. Nat Cell Biol.

[61] Hu JF, Yim D, Ma D, Huber SM, Davis N, Bacusmo JM (2021). Quantitative mapping of the cellular small RNA landscape with AQRNA-seq. Nat Biotechnol.

[62] Wang H, Huang R, Li L, Zhu J, Li Z, Peng C (2021). CPA-seq reveals small ncRNAs with methylated nucleosides and diverse termini. Cell Discov.

[63] Liu F, Clark W, Luo G, Wang X, Fu Y, Wei J (2016). ALKBH1-Mediated tRNA Demethylation Regulates Translation. Cell.

[64] Xu W, Yu M, Wu Y, Jie Y, Li X, Zeng X (2022). Plasma-Derived Exosomal SncRNA as a Promising Diagnostic Biomarker for Early Detection of HBV-Related Acute-on-Chronic Liver Failure. Front Cell Infect Microbiol.

[65] Ruggiero CF, Fattore L, Terrenato I, Sperati F, Salvati V, Madonna G (2022). Identification of a miRNA-based non-invasive predictive biomarker of response to target therapy in BRAF-mutant melanoma. Theranostics.

[66] Tong WG, Sandhu VK, Wood BL, Hendrie PC, Becker PS, Pagel JM (2015). Correlation between peripheral blood and bone marrow regarding FLT3-ITD and NPM1 mutational status in patients with acute myeloid leukemia. Haematologica.

