# Supplementary material for: Human circulating small non-coding RNA signature as a non-invasive biomarker in clinical diagnosis of acute myeloid leukaemia
Source: Theranostics. 2023 Feb 13;13(4):1289–301. doi: 10.7150/thno.80054 (PMC10008735; doi:10.7150/thno.80054)
Supplement: Supplementary file 1 — Supplementary figures and tables, methods. [file thnov13p1289s1.pdf]

Supplementary Material

Human circulating small non-coding RNA signature as a non-invasive biomarker in clinical diagnosis of acute myeloid leukaemia

Lin Xia[1*], Huanping Guo[1*], Xiao Wu[1*], Yinying Xu[1*], Pan Zhao[1*], Bingbing Yan[1], Yunjing Zeng[1], Yundi He[1], Dan Chen[1], Robert Peter Gale[2], Yunfang Zhang[3†], and Xi Zhang[1,4†].

[1]Medical Center of Hematology, Xinqiao Hospital, State Key Laboratory of Trauma, Burns and Combined Injury, Army Medical University, Chongqing, China.

[2]Haematology Centre, Department of Immunology and Inflammation, Imperial College London, London, UK.

[3]Clinical and Translational Research Center of Shanghai First Maternity and Infant Hospital, Shanghai Key Laboratory of Signaling and Disease Research, Frontier Science Center for Stem Cell Research, School of Life Sciences and Technology, Tongji University, Shanghai, China.

[4]Jinfeng Laboratory, Chongqing, China

* These authors contributed equally to this work.

[†]Corresponding author.

Correspondence: **Xi Zhang**, Medical Center of Hematology, Xinqiao Hospital, State Key Laboratory of Trauma, Burns and Combined Injury, Army Medical University, Chongqing, 400037, China; Jinfeng Laboratory, Chongqing, 401329, China. Tel: +86-023-68783496; Email: zhangxxi@sina.com; **Yunfang Zhang**, School of Life Sciences and Technology, Tongji University, Shanghai, 200092, China. Tel: +86-18810085579; Email: zhangyunfang@tongji.edu.cn.

Supplementary Methods

Ethics committee approval and patient consent

All experiments were performed in accordance with the principles set forth in the World Medical Association Declaration of Helsinki. This study was approved by the Institutional Ethics Committees of Xinqiao Hospital, and informed consent was signed by each participant.

Clinical data and sample collection

In total, 92 clinical samples including 80 preliminary diagnosis patients with de novo AML (AML) and 12 healthy controls (NC) from the Hematology Medical Center, Xinqiao Hospital, were enrolled in this study (**Table S1**). Peripheral blood samples from 50 AML patients and 12 healthy controls were collected. The peripheral blood samples and bone marrow samples from the same 30 AML patients were collected. Taken together, 122 samples containing either peripheral blood serum or bone marrow supernatant were prepared for small RNA library construction and high-throughput sequencing. All the subjects involved in this study were of Han Chinese descent. The diagnosis of AML patients was based on morphology, immunophenotyping, cytogenetics, and molecular genetics.

Peripheral blood serum and bone marrow supernatant isolation

5 ml peripheral blood from the median cubital vein was collected from each subject. 5 ml bone marrow was aspirated and collected from each participant under general anaesthesia. All the samples were placed at room temperature for 30 min to promote clotting. After blood clotting, the samples were centrifuged at 2,000 × g at 4 °C for 10 min to separate the serum. The isolated serums were

then transferred into new tubes and centrifuged again at the speed of 8,500 × g at 4 °C for 10 min to thoroughly remove cell debris contamination. All the samples were stored at -80 °C until RNA extraction.

Serum RNA extraction

Serum RNA was extracted by using TRIzol LS reagent (Invitrogen, Carlsbad, CA, USA) according to the manufacturer's protocol. Briefly, 0.25 ml serum was added to a 1.5 ml tube mixed with 0.75 ml TRIzol LS reagent and swirled. 1 μl cel-miR-39 was then added to the mixtures and vortexed vigorously. The mixtures were incubated on ice for 2 h with occasional vortexing to ensure that the serum was completely cracked. Then, 0.2 ml chloroform was then added to the mixtures, vortexed and incubated at room temperature for 10 min. The samples were centrifuged at 12,000 × g for 15 min at 4 °C. Next, the aqueous phase was collected into a tube filled with an equal volume of isopropanol. Then the mixtures were gently mixed with 1 μl glycogen (Invitrogen, Carlsbad, CA, USA), and refrigerated at −80 °C for at least 30 min to precipitate RNA. The mixture was centrifuged at 12,000 × g for 25 min at 4 °C, and the pellet was washed with 75% cold ethanol. Finally, the RNA pellet was dissolved in RNase-free water after being totally dried and stored at -80 °C for small RNA library construction, northern blot, Q-PCR, and RT-PCR.

Small RNA library construction and high-throughput sequencing

Small RNA library construction and sequencing were performed by BGI (Shenzhen, Guangdong, China). Briefly, total RNA was extracted from the PBS or BMS using TRIzol LS reagent (Invitrogen, Carlsbad, CA, USA), and qualified and quantified using a NanoDrop (NanoDrop, Madison, DC, USA) or Agilent

2100 bioanalyzer (Agilent, Santa Clara, USA). Subsequently, total RNA was separated by 15% urea denaturing polyacrylamide gel electrophoresis (PAGE), and small RNA regions corresponding to the 15-45 nt bands in the marker lane (14-30 ssRNA Ladder Marker) (Takara, Iwate, Japan) were excised and recovered. Then, the small RNAs were ligated to adenylated 3' adapters annealed to unique molecular identifiers (UMI), followed by the ligation of 5' adapters. The adapter-ligated small RNAs were subsequently transcribed into cDNA by Superscript II Reverse Transcriptase (Invitrogen, Carlsbad, CA, USA) and then several rounds of PCR amplification with PCR Primer Cocktail and PCR Mix were performed to enrich the cDNA fragments. The library was qualitative and quantitative in two ways: the distribution of the size of the fragments was verified using the Agilent 2100 bioanalyzer, and the library was quantified by real-time quantitative PCR (QPCR) (TaqMan Probe). The final ligation PCR products were sequenced using the DNBSEQ-G50 or Illumina-HiSeq 2500 platform.

Data processing and annotation for small-noncoding RNA sequencing data

To provide high-quality small noncoding RNA (sncRNA) profiles in healthy controls and AML patients, stringent criteria were adopted for data processing. Raw sequencing reads were processed using the software SPORTS 1.0 [1]. Following the SPORTS pipeline, Cutadapt [2] was first used to remove 5’ and 3’ adapters and discard sequencing reads either shorter than 15 nt or longer than 45 nt. Subsequently, the clean reads obtained were further aligned to the human reference genome (GRCH38/hg38), miRNA datasets (miRbase, v21), rRNA and YRNA datasets (NCBI nucleotide and gene database), genomic

tRNA datasets (GtRNAdb, v71, hg19), and mitochondrial tRNA datasets (mitotRNAdb, v72), piRNA datasets (piRBase, v29, and piRNABank, v30), and non-coding RNA datasets (Ensembl, release 89; Rfam, v12.3) to count the number of sncRNA transcripts by using Bowtie [3], without mismatch allowance. Finally, individual sncRNA expression levels were quantified and normalized to the total count of the reads that matched the genome of each individual sample, separately (RPM/CPM: reads/counts of exon model per million mapped reads). Additionally, tRNA-derived small RNAs (tsRNA) were grouped into four major categories, which are 5'tsRNA (derived from the 5' end of tRNA), inner'tsRNA (derived from the internal of tRNA), 3'tsRNA (derived from the 3' end of tRNA), and 3'CCA tsRNA (derived from the 3' end of mature tRNA). rRNA-derived small RNAs (rsRNA) were further classified into two major groups, nucleus-encoded rsRNA (cyto-rsRNA-5S/5.8S/18S/28S/45S) and mitochondria-encoded rsRNA (mt-rsRNA-12S/16S), according to the origination.

Detection of differentially expressed sncRNAs in AML

We performed comparative analysis of differentially expressed sncRNAs between healthy controls and AML patients using the R package edgeR (v3.36.0) [4]. Briefly, lowly expressed sncRNAs (counts of exon model per million mapped reads (CPM) < 1 in each individual sample) were removed. Sequencing depth and batch effect of both healthy controls and subtypes of AML patients were removed by the trimmed mean of M-values (TMM) method and dispersion was estimated using the quantile-adjusted conditional maximum likelihood method (qCML) method implemented in the package. Differentially expressed sncRNAs were analysed between healthy controls and AML patients using the exact test function, and multiple testing was corrected using the

Benjamini-Hochberg method with medium stringency. Using the parameters we mentioned above, a sncRNA was considered to be significantly differentially expressed between healthy controls and AML patients when the p value was <= 0.05 and the absolute value of log2 fold change was >= 1.

RT-PCR and quantitative RT-PCR

Reverse transcription for validation of sncRNA was performed as previously described [5] Briefly, serum total RNA from each subject was polyadenylated and then converted to cDNA using M-MuLV Reverse Transcriptase Reaction system (NEB, USA) with a unique adaptor. sncRNAs were amplified from cDNA using specific sncRNA primers (**Table S2**) in combination with the universal adaptor in a 10 µl reaction system containing 5 µl of GoTaq® Green Master Mix (Promega, Madison, WI, USA), 1 µl of cDNA template, 0.5 µl (10 µM) of each primer, and 3 µl of distilled water. The cycling conditions were conducted following GoTaq® Green Master Mix instructions. The PCR products were checked with 3% agarose gels. At least three healthy or AML individual PBS samples were used for RT-PCR validation of each target sncRNA. Meanwhile, the cDNA was also subjected to qRT-PCR. In the reaction tube,10 µl of GoTaq qPCR Master Mix (Promega, Madison, WI, USA), 1 µl (10 µM) of specific sncRNA primer, 1µl (10 µM) of universal adaptor, 1 µl of cDNA template, and 7 µl of distilled water were mixed and analysed by a CFX Connect Real-Time PCR Detection System (Bio-Rad, California, USA). In addition, cel-miR-39 was used as a spike-in control for normalization between samples. In total, 10 healthy control and 30-47 AML patient samples were used for quantitative RT-PCR validation of each target sncRNA.

Northern Blot

Northern blotting was performed as previously described [6] to verify the expression pattern of tsRNA (tsRNA-Gly^{CCC}) and ysRNA (ysRNA^{RNA4} and ysRNA^{RNY5}) in PBS of healthy controls. In brief, serum total RNA was electrophoresed by 15% urea-PAGE gel, and it was stained with SYBR Gold for imaging under a UV transilluminator. Then, RNA was transferred to Nytran Super Charged membranes (Roche, Switzerland) using TBE buffer (Invitrogen, Carlsbad, CA, USA) and UV cross-linked. The membrane was pre-hybridized with DIG pre-hybridization (Roche, Basel, Switzerland) and followed by incubation with DIG-labeled oligonucleotide probes (**Table S3**). After discarding the hybridization solution, the membrane was washed with low stringent buffer, high stringent buffer, and washing buffer in turn. Then, the membrane was blocked at room temperature for 3 h using blocking buffer (Roche, Basel, Switzerland). After that, the membrane was incubated again with DIG antibody (Roche, Basel, Switzerland) diluted in blocking buffer for another 1 h. The membrane was washed with DIG wash buffer, followed by incubation in developing buffer for 10 min. Finally, the membrane was stained with CSPD reagent (Roche, Basel, Switzerland) for 15 min in a dark environment at 37 °C and photographed with Bio-Rad system (California, USA).

Machine learning marker sncRNA discovery and performance evaluation

We experimented with the commonly used and highly efficient machine learning methods (Random Forest (v4.6-14) depicted in R) [7] to develop and evaluate binary classifiers based on several small noncoding RNA datasets. Our strategy for sncRNA signature selection was mainly divided into the following parts. First, differentially expressed miRNAs (242 in total) and tsRNAs (88 in total) (mentioned above) were selected to develop prediction models by using the

random forest with 1,000 randomizations. Subsequently, we further optimized a subset of 75-miRNAs and 39-tsRNAs as a diagnostic panel based on evidence of differential analysis and average expression level (differentially expressed in AML, expression level >= 10) for developing prediction models. To reduce the number of variables and improve model efficiency, we optimized a subset of 20-miRNAs and 19-tsRNAs as a diagnostic panel based on evidence of logistic regression models (differentially expressed in AML, expression level >= 10, and coefficients p-value <= 0.05) for the developing prediction models once again. In this study, we used two datasets involving different participant cohorts: 1) a cohort with 12 healthy individuals and 50 AML patient blood serum samples (62 samples in total, cohort-1, discovery cohort) and 2) a cohort with paired blood serum and bone marrow supernatant samples from 30 AML patients (60 samples in total, cohort-2, validation cohort). We applied the discovery cohort to perform the machine learning and cross-validation analyses. Briefly, with each iteration of the random forest model, one set of samples (n = 10) was randomly sampled and left out first, and the remaining samples (n = 52) were used as a training dataset for sncRNA modelling. Subsequently, the left-out samples were then used as an internal testing dataset for evaluating the prediction accuracy of sncRNA models. A total of 1,000 iterations were set and performed, and the averaged coefficient was used. In addition, the validation cohort was used as an independent cohort for validation. Additionally, the prediction error rates (out-of-bag errors) and the area under the receiver operating curve (AUC) were calculated to evaluate the performance of tsRNA or miRNA prediction models.

Correlation analysis of sncRNAs in PBS and BMS

To further determine the similarity of sncRNA expression profiles between PBS and BMS, we calculated pairwise correlations between PBS and BMS sncRNA expression profiles derived from individual AML samples. Pearson correlation coefficient (r), p values (p), and counts of expressed sncRNAs (n) were calculated by Pearson's correlation analysis.

Statistical analysis

Statistical significance for the analyses in this study was determined by Student's t test, one-way ANOVA, and two-way ANOVA with Fisher's LSD test. (*$p < 0.05$, **$p < 0.01$, ***$p < 0.001$, ****$p < 0.0001$, NS > 0.05)

Data sharing statement

Part of the sncRNA sequencing datasets is available in the Genome Sequence Archive for Human database (GSA-Human, Beijing Institute of Genomics, Chinese Academy of Sciences) under the accession number HRA001179.

Reference

1. Shi J, Ko EA, Sanders KM, Chen Q, Zhou T. SPORTS1.0: A Tool for Annotating and Profiling Non-coding RNAs Optimized for rRNA- and tRNA-derived Small RNAs. Genomics Proteomics Bioinformatics. 2018; 16: 144-51.
2. Martin M. Cutadapt removes adapter sequences from high-throughput sequencing reads. EMBnetjournal. 2011; 17: 10-2.
3. Langmead B, Trapnell C, Pop M, Salzberg SL. Ultrafast and memory-efficient alignment of short DNA sequences to the human genome. Genome Biol. 2009; 10: R25.
4. Robinson MD, McCarthy DJ, Smyth GK. edgeR: a Bioconductor package for differential expression analysis of digital gene expression data. Bioinformatics. 2010; 26: 139-40.
5. Zhang Y, Zhang Y, Shi J, Zhang H, Cao Z, Gao X, et al. Identification and characterization of an ancient class of small RNAs enriched in serum associating with active infection. J Mol Cell Biol. 2014; 6: 172-4.
6. Zhang Y, Zhang X, Shi J, Tuorto F, Li X, Liu Y, et al. Dnmt2 mediates intergenerational transmission of paternally acquired metabolic disorders through sperm small non-coding RNAs. Nat Cell Biol. 2018; 20: 535-40.
7. Breiman L. Random Forests. Machine Learning. 2001; 45: 5-32.

Supplementary Tables:

Table S1. Clinical characteristics of AML patients

Clinical characteristics	Count
**Gender [n (%)]**	
Female	34 (42.5)
Male	46 (57.5)
**Age at AML diagnosis [years, median (range)]**	52.5 (13-79)
**BM Blast at diagnosis (%, median (range))**	65 (18-97)
**PB WBC count at diagnosis (x10^9/L, median (range))**	10.15 (0.4-294.27)
**Karyotype [n (%)]**	
Normal karyotype	29 (36.25)
Abnormal karyotype	32 (40)
Not available	19 (23.75)
**AML subtypes [FAB, n (%)]**	
AML-M1	13 (16.25)
AML-M2	17 (21.25)
AML-M3	13 (16.25)
AML-M4	17 (21.25)
AML-M5	20 (25)
**Fusion gene [n (%)]**	
Positive	26 (32.5)
CBFβ -MYH11	3 (3.75)
MLL-AF9	1 (1.25)
PML-RARa	13 (16.25)
RUNX1-RUNX1T1	9 (11.25)
Negative	37 (46.25)
Not available	17 (21.25)
**CN AML-NPM1 [n (%)]**	
With *NPM1* mutation	6 (7.5)
Without *NPM1* mutation	37 (46.25)
Not available	37 (46.25)
**CN AML-*FLT3-ITD* [n (%)]**	
FLT3-ITD positive	8 (10)
FLT3-ITD negative	35 (43.75)
Not available	37 (46.25)
**CN AML-*ASXL1 or RUNX1* [n (%)]**	
With *ASXL1* or *RUNX1* mutation	10 (12.5)
Without *ASXL1* or *RUNX1* mutation	33 (41.25)
Not available	37 (46.25)
**Cytogenetic risk level at diagnosis [n (%)]**	
Favourable	24 (35.82)
Intermediate	18 (26.86)
Adverse	9 (13.43)
Not available	16 (23.88)

Table S2. Primer sequences RT-PCR and quantitative RT-PCR.

Name	Oligonucleotide sequences (5'- 3')
adaptor primer	GCGAGCACAGAATTAATACGACT CACTATAGGTTTTTTTTTTTTVN
3'universal reverse	GCGAGCACAGAATTAATACGACTC
**miRNA**	
mir-146a	TGAGAACTGAATTCCATGGGTT
mir-320a	AAAAGCTGGGTTGAGAGGGCGA
mir-451a	AAACCGTTACCATTACTGAGT
mir-122	TGGAGTGTGACAATGGTGTTT
**tsRNA**	
5'tsRNA$^{Gly\text{-}CCC}$	GCATTGGTGGTTCAGTGGTAGAATTC
5'tsRNA$^{Arg\text{-}CCT}$	GCCCCAGTGG CCTAA TGGAT AAGGC
inner'tRNA$^{Arg\text{-}CCT}$	CTAAGCCAGGGATTGTGGGT
3'tsRNA$^{Arg\text{-}CCT}$	TCGAGTCCCACCTGGGGTACCA
5'tsRNA$^{Ser\text{-}AGA}$	GTAGTCGTGGCCGAGTGGTTAA
inner'tsRNA$^{Ser\text{-}AGA}$	GACTAGAAATCCATTGGGGTCT
3'tsRNA$^{Ser\text{-}AGA}$	GGTTCGAATCCTGCCGACTACG
5'tsRNA$^{Thr\text{-}CGT}$	GGCGCGGTGGCCAAGTGGTAA
inner'tsRNA$^{Thr\text{-}CGT}$	TAAGGCGTCGGTCTCGTAAACC
3'tsRNA$^{Thr\text{-}CGT}$	GGTTCGAACCCCGTCCGTGCCT
inner'tsRNA$^{Leu\text{-}CAA}$	ACCTTGCCTGCGGGCTTTCTGGTCT
5'tsRNA$^{Lys\text{-}CTT}$	GCCCGGCTAGCTCAGTCGGTAGAGC
5'tsRNA$^{Val\text{-}CAC}$	GCTTCTGTAGTGTAGTGGTTATCAC
5'tsRNA$^{Cys\text{-}GCA}$	GGGGGTATAGCTCAGTGGTAGAGCA
**ysRNA**	
ysRNA^{RNY4}	GGCTGGTCCGATGGTAGTGGGTTAT
ysRNA^{RNY5}	AGTTGGTCCGAGTGTTGTGGGTT
**rsRNA**	
rsRNA-5S	GTCTACGGCCATACCACCCTGAACG
rsRNA-5.8S	CGACTCTTAGCGGTGGATCACTC
rsRNA-18S-#1	CTCGTAGTTGGATCTTGGG
rsRNA-18S-#2	TTTCGGAACTGAGGCCATGATTA
rsRNA-18S-#3	AAGGAATTGACGGAAGGGCACCACCAGG
rsRNA-18S-#4	ACGGCCCTGGCGGAGCGCTGAGAAG
rsRNA-28S-#1	AGGAGTCTAACACGTGCGCG
rsRNA-28S-#2	AGCAGGACGGTGGCCATGGA
rsRNA-28S-#3	AGGGCTGGGTCGGTCGGGCTGGGGCG

Table S3. DIG-labeled oligonucleotide probes for Northern blot.

Name	Oligonucleotide sequences (5’- 3’)
**tsRNA**	
5'tsRNA$^{Gly\text{-}CCC}$	GAATTCTACCACTGAACCACCAATGC
inner'tsRNA$^{Gly\text{-}CCC}$	CCGGGTCTCCCGCGTGGGAGGCGAG
3'tsRNA$^{Gly\text{-}CCC}$	TGGTGCATTGGCCGGGAATTGAAC
**ysRNA**	
ysRNA^{RNY4}	ATAACCCACTACCATCGGACCAGCC
ysRNA^{RNY5}	AACCCACAACACTCGGACCAACT

Supplementary Figures

Figure S1

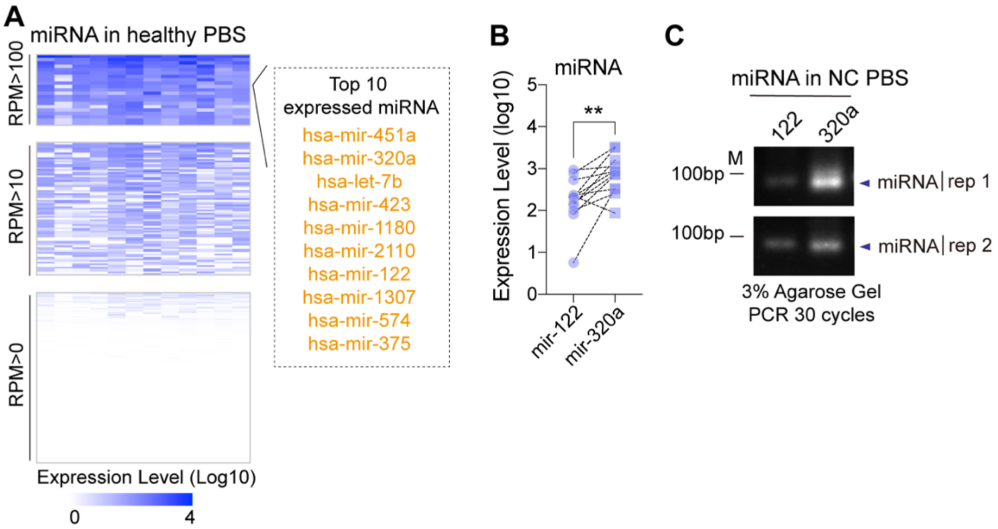

Figure S1. miRNA profiles in healthy human PBS. (A) Expression heatmap of miRNA in human PBS. (B) Expression levels of mir-122 and mir-320a in human PBS. (C) Validation of mir-122 and mir-320a by RT-PCR, at least three healthy individual PBS samples were used for validation.

Figure S2

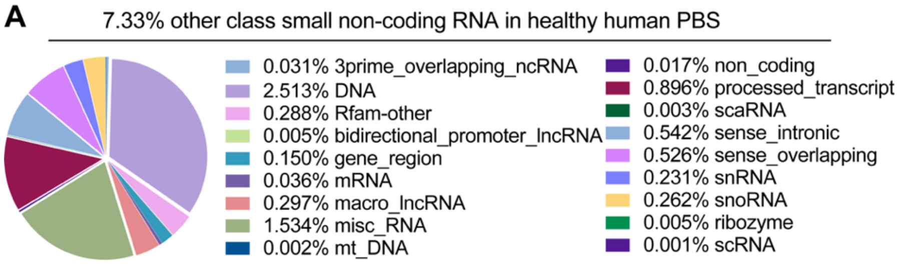

Figure S2. Relative proportion of sncRNAs belonging to the 'other' group in healthy human PBS. (A) Relative proportion of sncRNAs belonging to the 'other' group in human PBS.

Figure S3

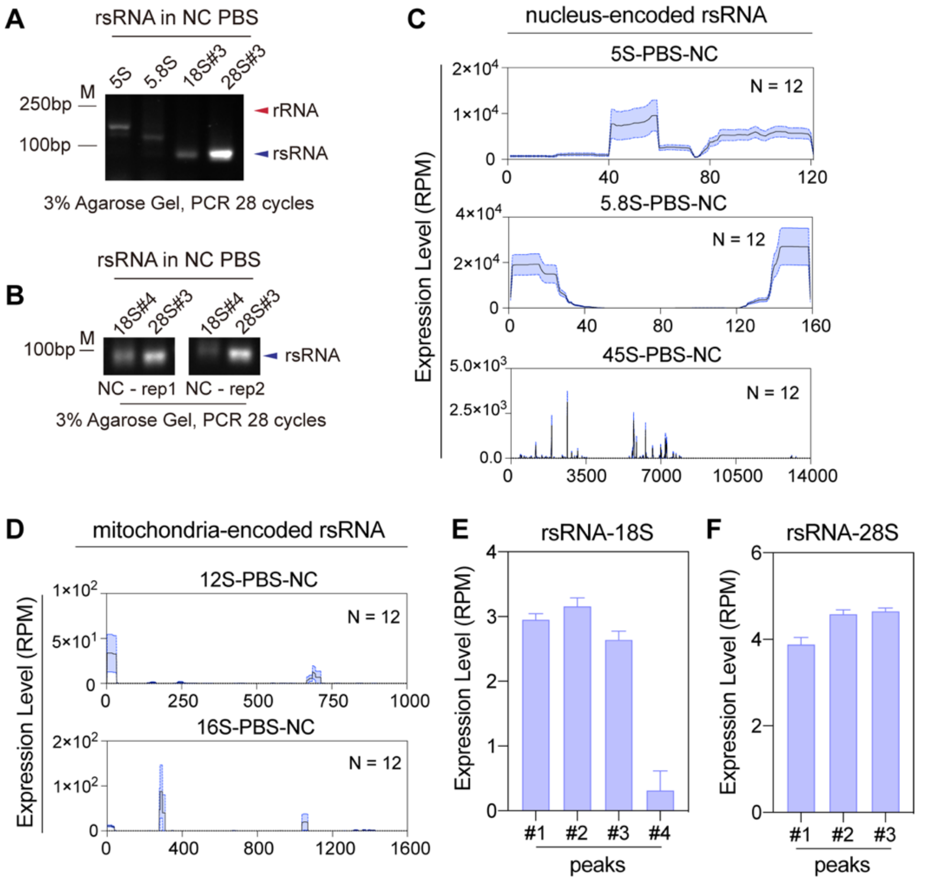

Figure S3. Characterization of rsRNA-generating loci in healthy human PBS. (A-B) Validation of rsRNA including 5S, 5.8S, 18S#3 and 28S#3 by RT-PCR, at least three healthy individual PBS samples were used for validation. (C-D) Expression signature and sequence mapping location of rsRNA in human PBS on their parental rRNA, including (C) nucleus-encoded (5S, 5.8S, and 45S) rRNA and (D) mitochondria-encoded (12S and 16S) rRNA. Expression levels are presented as means ± SEM. (E-F) The expression level of rsRNA-18S and rsRNA-28S peaks, the expression level was evaluated by RPM, reads of exon model per million mapped reads.

Figure S4

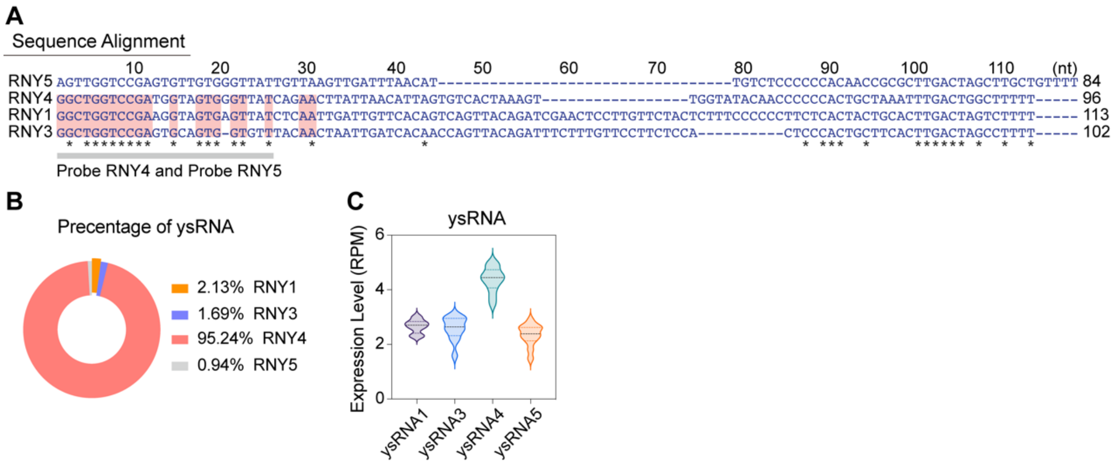

Figure S4. Profiling of ysRNA features in healthy human PBS. (A) Multiple sequence alignment of four human YRNAs, including RNY1, RNY3, RNY4, and RNY5. (B) Proportion distribution of four ysRNA categories in healthy human PBS. (C) Comparison of relative expression levels of four ysRNA categories in healthy human PBS.

Figure S5

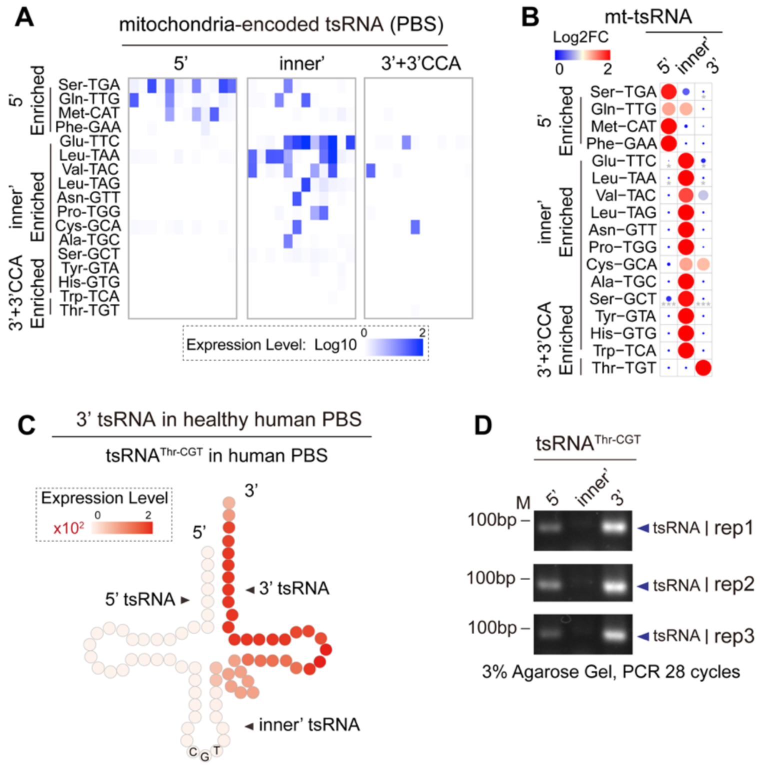

Figure S5. Identification of tsRNA signatures in healthy human PBS. (A-B) Expression heatmap of mt-encoded tsRNA in healthy human PBS. (C) Schematic illustration at the single-base resolution for tsRNA enrichment on tRNA$^{Thr-CGT}$ in PBS. (D) Validation of 5', inner', and 3'tsRNA$^{Thr-CGT}$ by RT-PCR, at least three healthy individual PBS samples were used for validation.

Figure S6

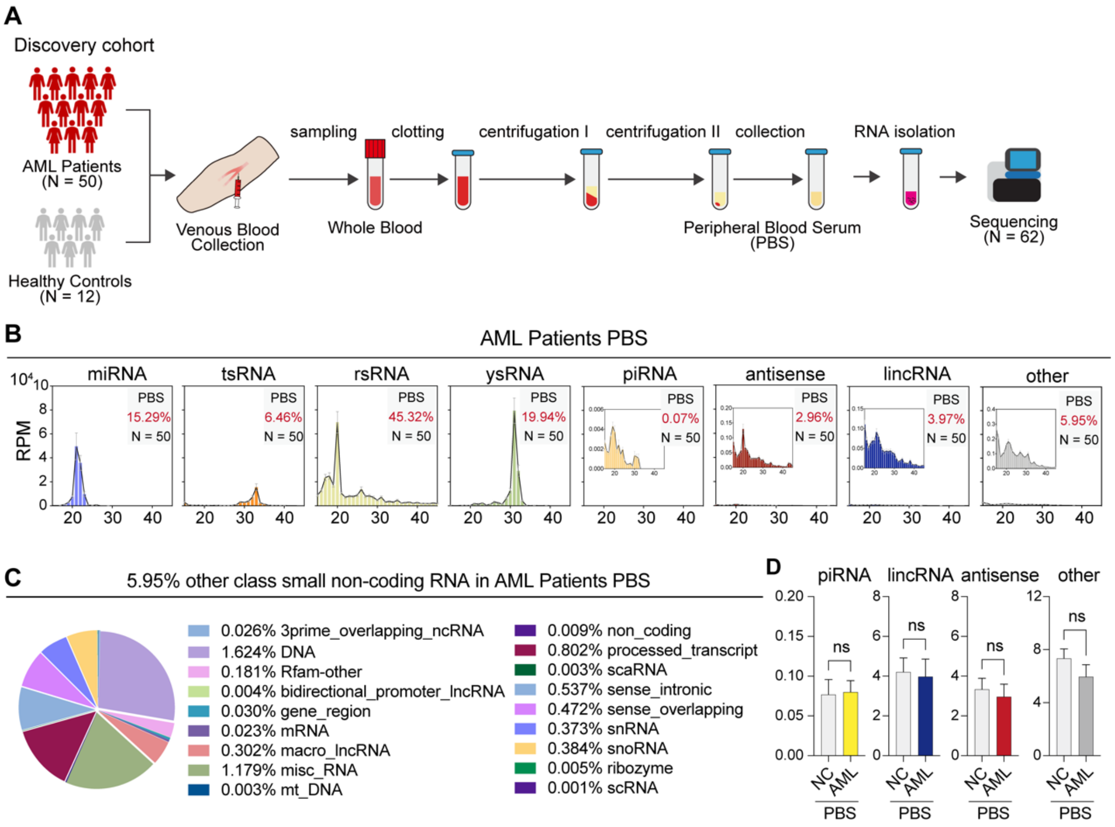

Figure S6. Altered landscape of sncRNAs in the PBS of AML patients. (A) Schematic overview of the experimental design. In the discovery cohort, 62 libraries of PBS from 12 healthy controls and 50 AML patients were profiled by sncRNA-seq. (B) Length distribution of selected types of sncRNAs in the PBS of AML patients (n = 50). The ‘other’ group contains sncRNAs derived from miscRNA, snoRNA, snRNA, and processed transcript, etc. (C) Relative proportion of sncRNAs belonging to the ‘other’ group in PBS of AML patients. (D) Comparison of piRNA, lincRNA, antisense, and the ‘other’ group in PBS between healthy controls and AML patients.

Figure S7

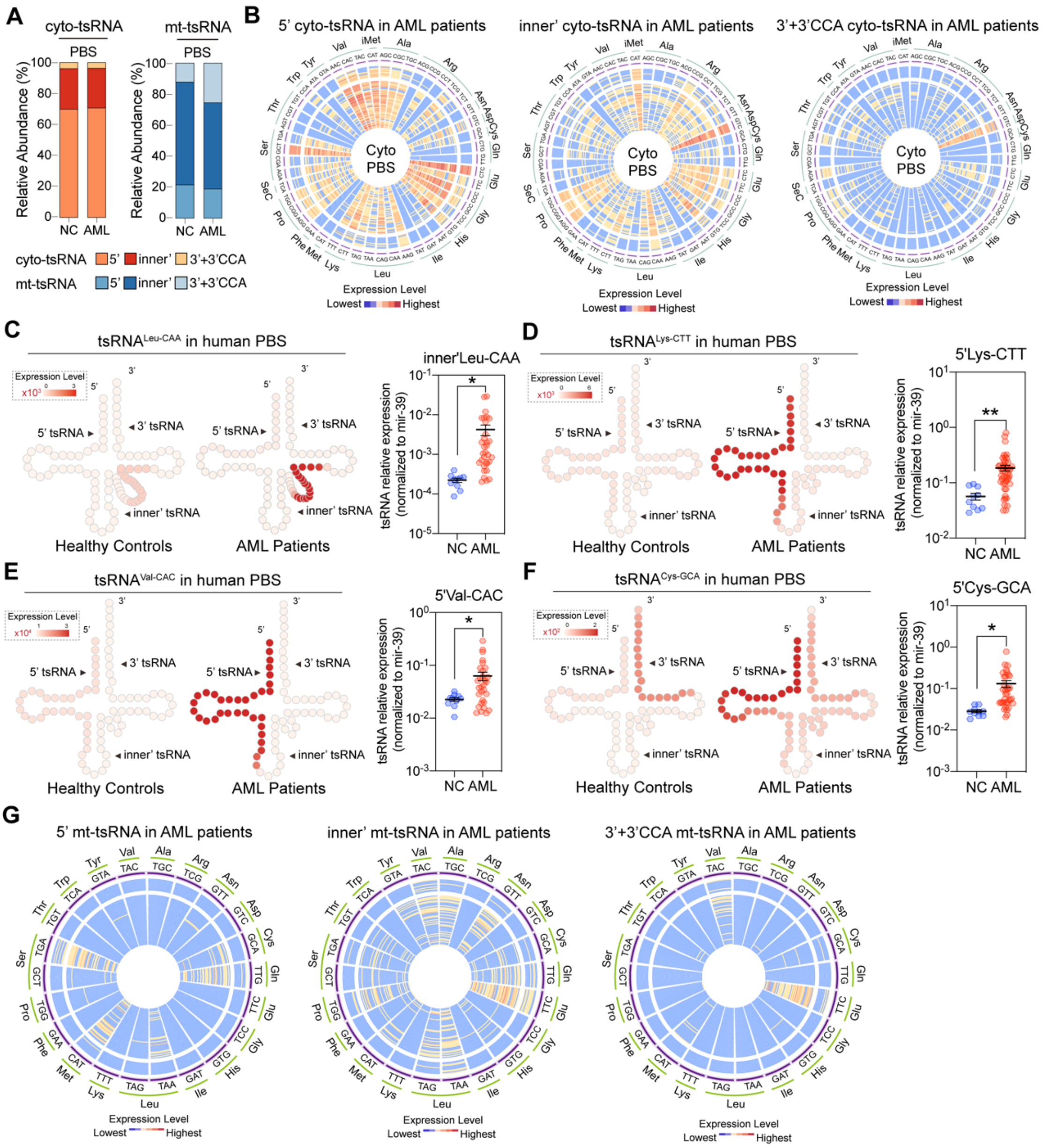

Figure S7. Characteristics of differentially expressed circulating tsRNAs in AML. (A) Comparison of the overall proportion distribution of four distinct tsRNA categories (5’tsRNA, inner’tsRNA, 3’+3’CCA tsRNA) for nucleus-encoded tsRNA (left) and mitochondria-encoded tsRNA (right) in PBS among healthy controls (n = 12) and AML patients (n = 50). (B) Expression level comparison of nucleus-encoded 5’tsRNA, inner’tsRNA, and 3’+3’CCA tsRNA that categorized by the anti-codon of an amino acids between healthy controls

and AML patients in PBS. The outer circles (n = 12) represent healthy controls, and the inner circles (n = 50) indicate AML patients. Each circle represents one sample. (C-F) Schematic illustration at the single-base resolution and quantitative RT-PCR validation for differentially expressed tsRNA: (C) tsRNA$^{Leu\text{-}CAA}$, (D) tsRNA$^{Lys\text{-}CTT}$, (E) tsRNA$^{Val\text{-}CAC}$ and (F) tsRNA$^{Cys\text{-}GCA}$. (G) Expression level comparison of mitochondria-encoded 5'tsRNA, inner'tsRNA, and 3'+3'CCA tsRNA that categorized by the anticodon of an amino acid between healthy controls and AML patients in PBS. The outer circles (n = 12) represent healthy controls, and the inner circles (n = 50) indicate AML patients. Each circle represents one sample.

Figure S8

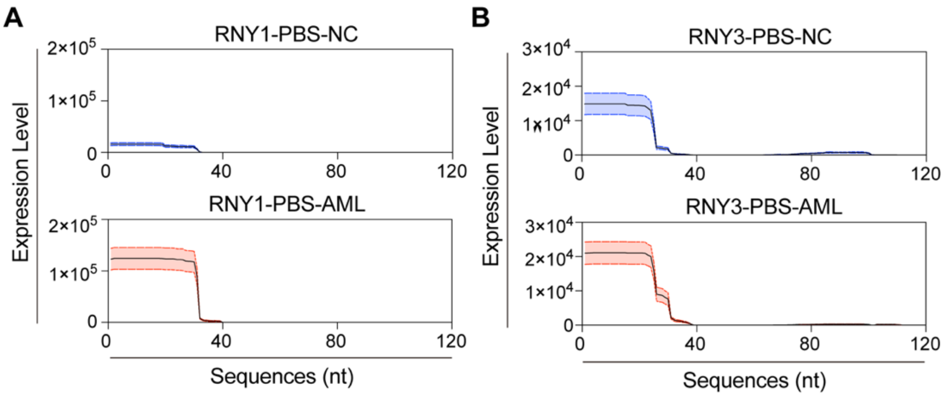

Figure S8. Identification of ysRNA variation between healthy controls and AML patients. (A-B) Expression signature and sequence mapping location of ysRNA^{RNY1} and ysRNA^{RNY3} in human PBS on their parental YRNAs. Expression levels are presented as means ± SEM.

Figure S9

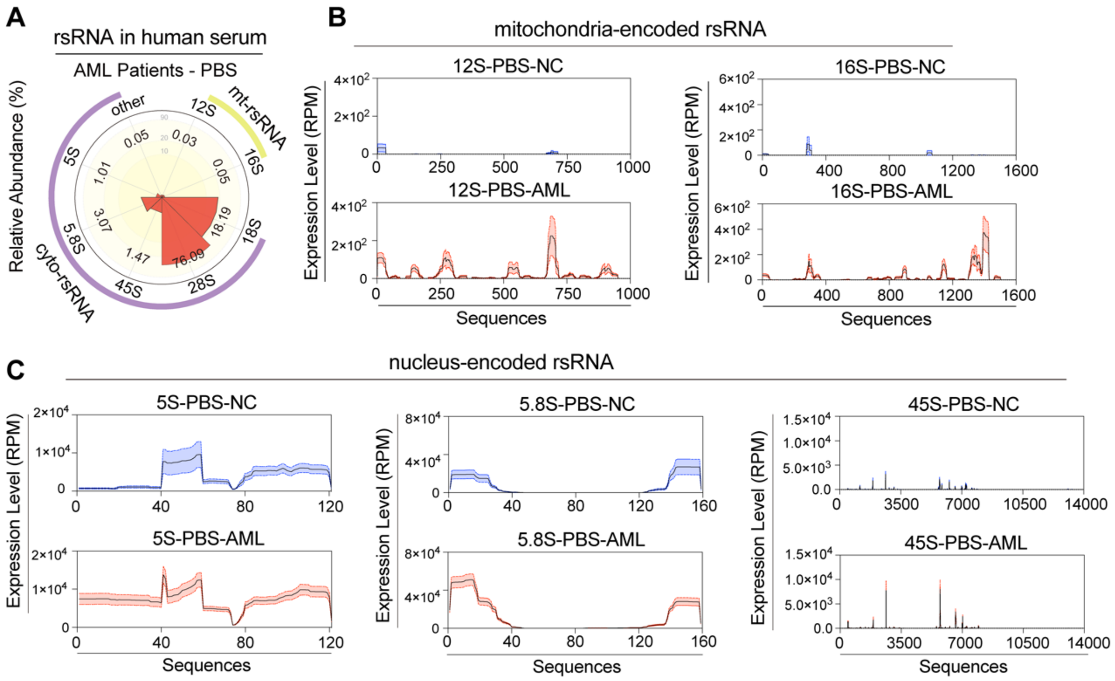

Figure S9. Characterization of the rsRNA signature in AML patients. (A) Distribution of five nucleus-encoded rsRNA (cyto-rsRNA: 5S, 5.8S, 18S, 28S, and 45S) and two mitochondria-encoded rsRNA (mt-rsRNA: 12S and 16S) categories in AML patients. (B) Comparison of rsRNA-generating location by rsRNA sequencing data on two mitochondria-encoded rsRNAs (12S and 16S) among healthy controls and AML patients in PBS. (C) Comparison of rsRNA-generating location by rsRNA sequencing data on three nucleus-encoded (5S, 5.8S, and 45S) rRNAs among healthy controls and AML patients in PBS.

Figure S10

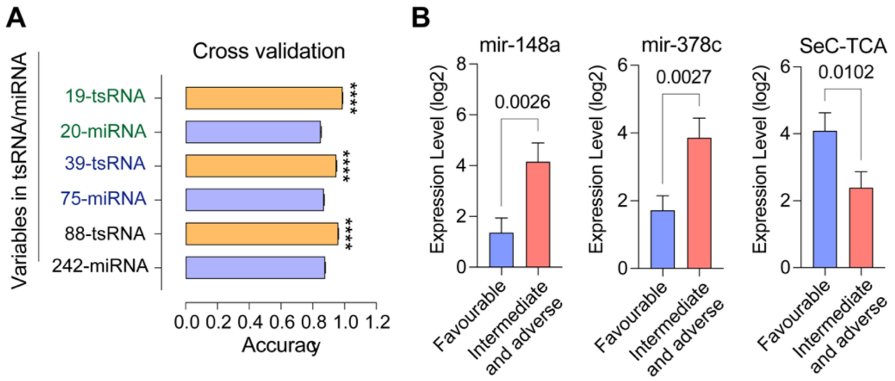

Figure S10. tsRNA-based feature screening robustly discriminates subjects with AML from controls. (A) Performance of distinct miRNA and tsRNA datasets in the discovery cohort. (B) Expression level of several sncRNAs at newly diagnosed correlated with risk levels.

Figure S11

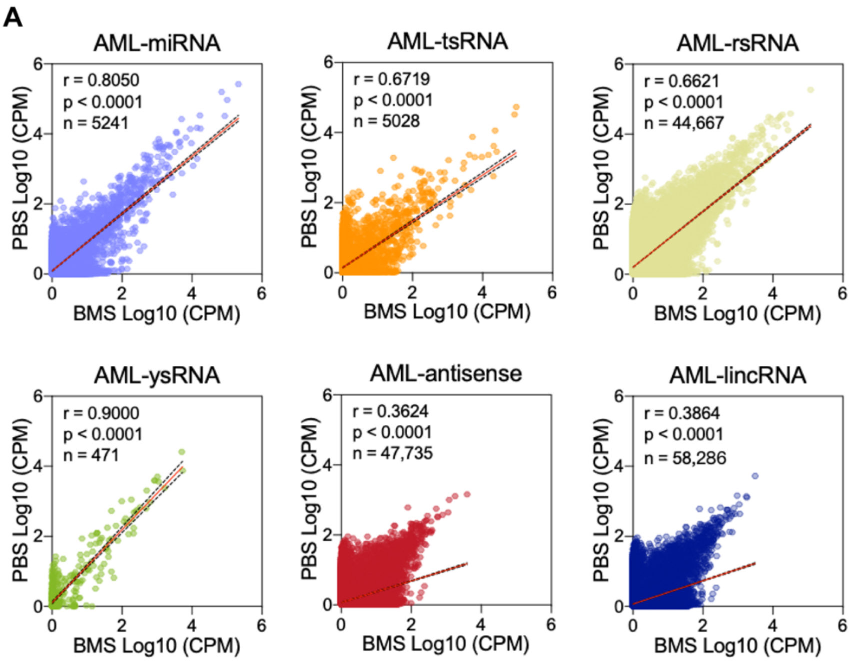

Figure 11. Correlation analysis of sncRNA expression levels between PBS and BMS in AML patients. (A) Scatter plot shows the correlation of miRNA, tsRNA, rsRNA, ysRNA, antisense, and lincRNA expression profiles between paired PBS and BMS derived from an individual in the AML group.
